# Conserved Protein–Polymer Interactions across Structurally Diverse Polymers Underlie Alterations to Protein Thermal Unfolding

**DOI:** 10.1021/acscentsci.2c01522

**Published:** 2023-03-14

**Authors:** Amanda Pritzlaff, Guillaume Ferré, Elia Dargassies, Crystal O. Williams, Daniel D. Gonzalez, Matthew T. Eddy

**Affiliations:** Department of Chemistry, University of Florida, 126 Sisler Hall, Gainesville, Florida 32611, United States

## Abstract

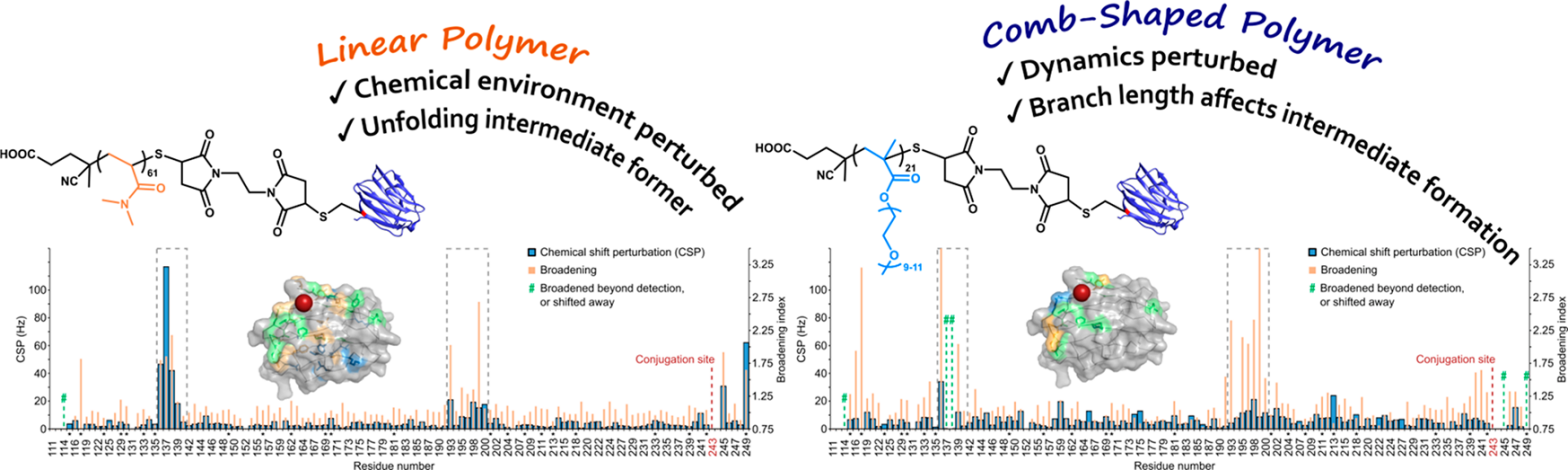

Protein–polymer conjugates are widely used in many clinical and industrial applications, but lack of experimental data relating protein–polymer interactions to improved protein stability prevents their rational design. Advances in synthetic chemistry have expanded the palette of polymer designs, including development of nonlinear architectures, novel monomer chemical scaffolds, and control of hydrophobicity, but more experimental data are needed to transform advances in chemistry into next generation conjugates. Using an integrative biophysical approach, we investigated the molecular basis for polymer-based thermal stabilization of a human galectin protein, Gal3C, conjugated with polymers of linear and nonlinear architectures, different degrees of polymerization, and varying hydrophobicities. Independently varying the degree of polymerization and polymer architecture enabled delineation of specific polymer properties contributing to improved protein stability. Insights from NMR spectroscopy of the polymer-conjugated Gal3C backbone revealed patterns of protein–polymer interactions shared between linear and nonlinear polymer architectures for thermally stabilized conjugates. Despite large differences in polymer chemical scaffolds, protein–polymer interactions resulting in thermal stabilization appear conserved. We observed a clear relation between polymer length and protein–polymer thermal stability shared among chemically different polymers. Our data indicate a wide range of polymers may be useful for engineering conjugate properties and provide conjugate design criteria.

## Abstract 2

Integrative biophysical data reveal similar protein−polymer interactions underlie improved protein thermal stability, providing a blueprint to design conjugates with improved properties.

## Introduction

Engineering proteins by chemical conjugation with polymers is an effective strategy to improve the robustness of proteins, a critical advantage for their use as biologics and in industrial applications.^1,2^ This strategy underlies the use of PEGylation to develop over 30 FDA-approved biologics, many of which are used in treating cancers and inflammatory diseases.^3^ The development of protein–polymer conjugates is largely empirical, and designing protein–polymer conjugates with predictable chemical properties remains a grand challenge. Critical to addressing this challenge is the need for improved molecular understanding of protein–polymer interactions that give rise to beneficial properties of conjugates such as improved thermal stability.^4,5^

Poly(ethylene glycol) (PEG) is widely used to conjugate biological macromolecules, including proteins^6,7^ and the lipids used in mRNA vaccines.^8^ However, the ubiquitous presence of PEG in consumer products and drugs has increased the prevalence of anti-PEG antibodies,^9,10^ which can cause premature drug release and degradation.^11^ This has motivated the need to explore new polymer chemical scaffolds that provide the benefits of conjugation while avoiding immune system detection. Advances in polymer synthesis have expanded the chemical architectures and properties of polymers used for protein–polymer conjugates beyond the linear PEG scaffold.^12,13^ However, more experimental data are needed that relate polymer architectures, degree of branching, and polymer lengths to protein–polymer interactions that provide favorable protein stability.

We prepared biomedically relevant, PEG-alternative polymers to systematically investigate the properties of polymers conjugated with the carbohydrate recognition domain of the human galectin protein Gal3 (Gal3C). The polymers poly(oligoethylene glycol methacrylate) (POEGMA) and poly(dimethylacrylamide) (PDMA) were synthesized with radical addition–fragmentation chain transfer (RAFT) polymerization.^14,15^ PDMA and POEGMA were chosen as polymers for conjugation due to their reported advantages as PEG alternatives. These advantages include improved plasma circulation times,^16^ a decrease in anti-PEG antibody formation,^17^ and an increased ability to evade detection by the immune system.^18^ We leveraged the RAFT synthesis approach to generate a series of each polymer with varying degrees of polymerization (*n*) and branch length (*m*), to delineate the contributions of these polymer characteristics to the properties of conjugated Gal3C.

**Figure 1:**
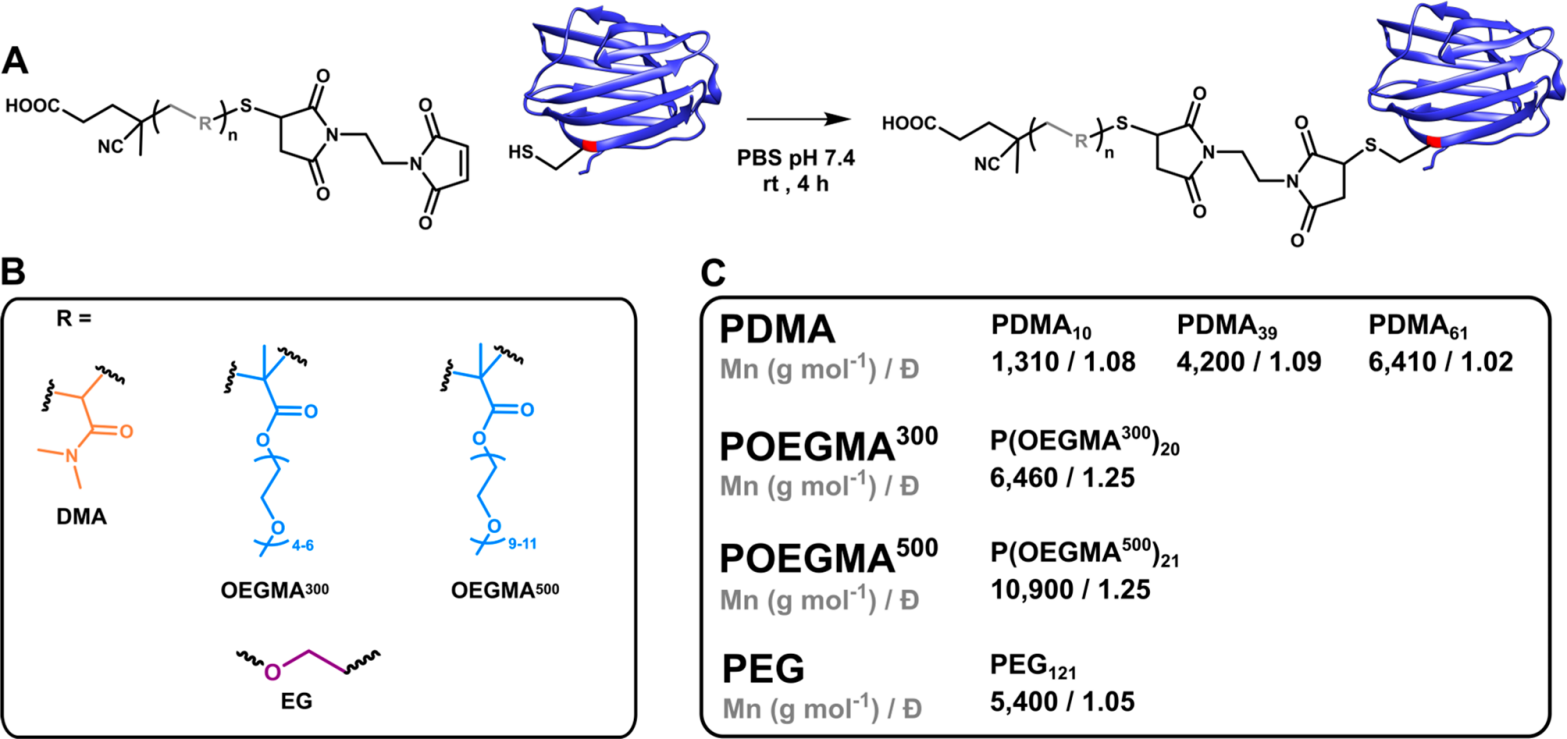
Properties of polymers used to generate conjugates with Gal3C[T243C] and general conjugation scheme. (A) Grafting-to conjugation scheme for generating Gal3C[T243C]–polymer conjugates using RAFT-generated polymers. (B) Chemical structures of monomers dimethylacrylamide (DMA) and oligoethylene glycol methacrylate (OEGMA) of number-averaged molecular weights (*M*_n_’s) of 300 and 500 g/mol used to synthesize the polymers. Ethylene glycol (EG) derived poly(ethylene glycol) (PEG) is shown for comparison. (C) Final *M*_n_ values and dispersities (*Đ*’s) of polymers conjugated to Gal3C[T243C] as obtained by SEC.

POEGMA and PDMA are among the most promising PEG alternatives in bioconjugation.^19−23^ The documented physical properties of the polymers provide a basis for testing properties of their corresponding protein conjugates. Both PDMA and POEGMA are neutral polymers, but they exhibit significantly different architectures and hydrophobiticites (Figure 1). Similar to PEG, PDMA is a hydrophilic, linear polymer with a random coil conformation in solution.^24^ POEGMA is a “comb-shaped” polymer with a hydrophobic methacrylate backbone and amphiphilic oligo(ethylene glycol) side chains, resulting in tunable thermal transitions.^25^ Increased branching (*m*) correlates with an increased cloud point temperature, as the longer branches are thought to better shield the polymer backbone from dehydration and self–self-hydrophobic interactions.^22,26^

For the present study, POEGMA^300^ (*m* = 4–6, *n* = 20) and POEGMA^500^ (*m* = 9–11, *n* = 21) were selected. This selection was motivated by earlier work describing α-chymotrypsin–POEGMA conjugates which, at *n* = 20, exhibited cloud points of ∼35 °C for *m* = 4–6 and ∼80 °C for *m* = 9–11.^27^ This indicated that polymers with a known thermal transition alter protein thermal properties. Other reports of POEGMA or PDMA conjugate properties vary, ranging from enhancement of desirable properties,^20,23,28,29^ to no benefit,^30^ or to diminishment.^21,31,32^ Thus, more experimental data are needed to predict beneficial configurations of PDMA or POEGMA in protein conjugates. To provide an atomic-level view of the impact of POEGMA and PDMA conjugated to Gal3C, we used nuclear magnetic resonance (NMR) spectroscopy in aqueous solutions to view changes in the protein backbone upon conjugation. Highly resolved NMR spectra of both conjugates facilitated residue-specific observations of the protein backbone structure and dynamics upon conjugation. Concurrently, we determined the thermal melting profiles of the same conjugates used for NMR and for a wider range of POEGMA and PDMA polymers with varying chain lengths and degrees of polymerization. By systematically varying these properties, we could develop a set of guidelines for preparing samples of conjugated Gal3C with more robust thermal melting profiles. Comparison of these data with earlier studies with PEG allowed us to evaluate the potential effectiveness of using alternative polymers in designing protein–polymer conjugates.

## Results and Discussion

### Polymer Synthesis and Characterization

Protein–polymer conjugates were generated with a grafting-to scheme in which a complete polymer was covalently attached to Gal3C[T243C] using thiol-Michael addition (Figure 1A). The grafting-to method was selected to ensure mild conjugation conditions that preserved Gal3C structure and function, despite the drawback of sterically limited conversion. RAFT polymerization (Scheme S1A) was used to generate five different polymers for conjugation with the following three monomers: dimethylacrylamide (DMA) and two molecular weights of oligoethylene glycol methacrylate (OEGMA^300^ and OEGMA^500^), which have structures shown in Figure 1B. The ethylene glycol (EG) monomer of PEG is also included for comparison in Figure 1B.

A total of five polymers were synthesized to interrogate the effects of architecture, degree of polymerization (*n*), and side chain length (*m*) on conjugate properties. All polymer number-averaged molecular weights (*M*_n_) and polydispersity indices (*Đ*) were characterized by size exclusion chromatography (SEC) (Figure 1C). Values for the PEG-maleimide polymer, which was previously conjugated to Gal3C[T243C] with a similar approach, are also included. The size exclusion chromatograms for each RAFT polymer are shown in Figure S1. First, P(OEGMA^500^)_21_ and PDMA_61_ polymers were synthesized by targeting molecular weights that would result in SEC elution times comparable to that of the PEG polymer previously conjugated to Gal3C (5400 g/mol PEG-maleimide) (Figure S2).^33^ With this approach, hydrodynamic size was used as a control between the two polymers with different architectures and the previously studied PEG.

### RAFT Polymerization of Polymer Series Prior to Functionalization

To study the effect of PDMA degree of polymerization (*n*) on Gal3C properties, intermediate-sized PDMA_10_ and PDMA_39_ were synthesized (Figure S1A,B). To interrogate the effect of side chain length of comblike polymers on conjugate properties, a second OEGMA-based polymer with shorter side chains, P(OEGMA^300^)_20_, was synthesized (Figure S1E) at the approximate degree of polymerization (*n*) of P(OEGMA^500^)_21_. Though the PDMA series featured lower dispersity as compared to the POEGMA series (Figure 1C), SEC data indicated that all polymers were monodispersed before functionalization (Figure S1). Assigned ^1^H NMR spectra of each polymer confirmed their chemical identities (Figures S3 and S4).

### Polymer End-Group Modification

To rapidly functionalize the RAFT polymers with cysteine-reactive maleimide groups, a two-step end-group-functionalization scheme was employed (Scheme S1B,C). Aqueous desalting columns allowed rapid purification of small batches of polymer after each synthetic step. First, the trithiocarbonate end group was cleaved using hydrazine aminolysis to reveal a thiol group (Scheme S1B) following a modified procedure from Shen et al.^34^ UV–vis spectra indicated that the trithiocarbonate peak at 315 nm disappeared after reacting with hydrazine, indicating near-quantitative conversion for each polymer (Figure S5). The polymeric thiols were then functionalized with protein-reactive maleimide (Scheme S1C) by reacting with bismaleimide linker (**1**); linker structure was verified by ^1^H NMR (Figure S6 and Figures S3 and S4, blue traces) and purified via a desalting column. SEC data (Figure S1, red traces) and ^1^H NMR spectra (Figures S3 and S4, green traces) were used to characterize the final maleimide-functionalized polymers. After functionalization with **1**, each POEGMA polymer ^1^H NMR spectrum featured a doublet in the maleimide region at 6.72 ppm, which was integrated against the OEGMA monomer methyl peaks (3.35 ppm) and corresponded with 21% (POEGMA^500^_21_-mal) and 12% (POEGMA^300^_20_-mal) conversion to maleimide end group (Figure S3A,B, green traces). The size exclusion chromatograms postfunctionalization (Figure S1D,E) were monomodal, as expected for end-group functionalization with the bismaleimide linker (**1**). Additionally, the postfunctionalization SEC data of the POEGMA polymers showed a slight shift to higher elution times, indicating that most of the sample is the lower molecular weight polymeric thiol, as expected with only 12–21% functionalization of the bismaleimide.

As compared to the methacrylate POEGMA polymers, the acrylamide PDMA polymer series featured some polydispersity post-end-group modification. The two smallest PDMA polymers (PDMA_10_-mal and PDMA_39_-mal) featured multimodal SEC chromatograms postfunctionalization with **1** (Figures S1A,B, respectively). These polymers were conjugated to Gal3C[T243C] without further purification because only the monomeric polymer species was expected to be protein-reactive. Postfunctionalization, the PDMA_61_-mal chromatogram featured a monomodal peak with an elution time similar to that of polymer prefunctionalization (Figure S1C). As with the chromatograms of POEGMA postfunctionalization, PDMA_61_ exhibited a slight shift to a higher elution time due to the predominant product being the lower molecular weight polymeric thiol. The PDMA_61_-mal ^1^H NMR spectrum (Figure S4A, green trace) featured a peak in the maleimide region at 6.62 ppm, and rough integration against the polymer *N*-methyl peaks (2.9 ppm) yielded a conversion of 8%. Due to the multimodal chromatograms observed for PDMA_10_-mal and PDMA_39_-mal, the ^1^H NMR spectra were provided without full assignment (Figure S4B,C, green traces). A ^1^H NMR spectrum of PDMA_39_-mal revealed a peak in the expected maleimide region at 6.67 ppm, indicating the presence of the proper end-group modification in one product. PDMA_10_-mal was found to be the most polydispersed by SEC analysis, and a distinct maleimide peak was not resolved in its ^1^H NMR spectrum, suggesting a lower percentage of monomeric polymer-maleimide compared to other products in the mixture. Despite this, the product mixture was still reacted with Gal3C[T243C] as large excess equivalents of the polymer were used, allowing for observed conjugate formation even though the desired end group was a minor product.

### Grafting-To Conjugation with Gal3C[T243C]

The polymers were conjugated to Gal3C[T243C] with a grafting-to scheme (Figure 1A). An excess of each aqueous polymer stock was used, with 10 equiv of reactive polymer-maleimide present to participate in a thiol-Michael addition with the solvent-exposed cysteine, C243, over the course of 4 h. Previously, it was determined that only C243 was functionalized with 5400 g/mol PEG-maleimide under similar grafting-to conditions with a shorter reaction time, and thus we anticipated site specificity would be preserved.^33^

To mimic conditions used for conjugating RAFT-synthesized polymers herein, a control reaction using excess PEG-maleimide (10 equiv) was performed to assess for multiple additions of PEG after 4 h at room temperature (Figure S7). A reaction time of 4.5 h showed mono-PEGylated Gal3C[T243C] as the primary product; only a minor amount of a higher molecular weight side product was detected with SEC (Figure S7B) and SDS-PAGE at several crude reaction time points (Figure S7C). MALDI-TOF revealed that this side product had a mass near the expected mass of a conjugate dimer (Figure S7D). This dimer side product was hypothesized to be the result of contamination of manufacturer monomethoxy-PEG with PEG-diol.^1,35^ Overall, the vast majority of the product is Gal3C[T243C] with one addition of the polymer. This suggested that conjugation with non-PEG polymers would also result in single polymer attachments at site C243 in Gal3C[T243C].

### Gal3C[T243C] Conjugate Purification and Assessment of Ligand Binding Function

**Figure 2:**
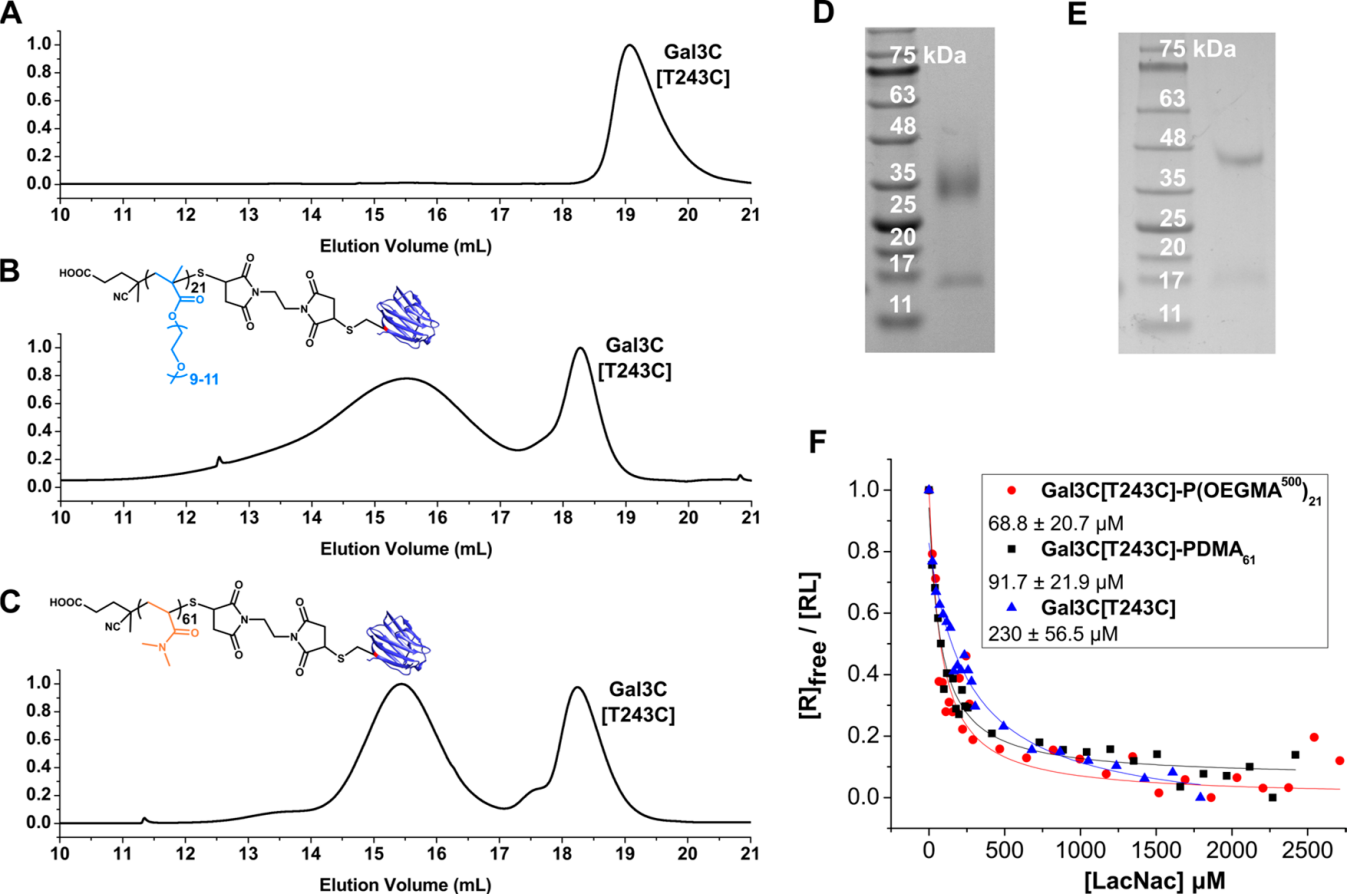
Preparation and equilibrium binding affinities of Gal3C[T243C]–P(OEGMA^500^)_21_ and Gal3C[T243C]–PDMA_61_ conjugates compared to unmodified Gal3C[T243C]. (A) Reference chromatogram of unmodified Gal3C[T243C]. (B and C) SEC purification of (B) Gal3C[T243C]–P(OEGMA^500^)_21_ and (C) Gal3C[T243C]–PDMA_61_. (D and E) SDS-PAGE of representative conjugate samples for (D) Gal3C[T243C]–P(OEGMA^500^)_21_ and (E) Gal3C[T243C]–PDMA_61_. For both gels, the left lane is an annotated molecular weight marker. (F) Equilibrium binding of LacNac to Gal3C[T243C]–P(OEGMA^500^)_21_, Gal3C[T243C]–PDMA_61_ and Gal3C[T243C] measured with intrinsic tryptophan fluorescence. Dissociation constants (*K*_D_’s) for LacNac are reported in the figure legend as an average of three replicates ± standard deviation. A single replicate with a *K*_D_ closest to the average is plotted for each construct.

Gal3C[T243C] was first conjugated with the PEG analogues P(OEGMA^500^)_21_ and PDMA_61_, and conjugate protein function was assessed (Figure 2). These two polymers were chosen to interrogate the effect of polymer architecture on conjugate properties and were designed to exhibit hydrodynamic size similar to that of 5400 g/mol PEG (Figure S2). The conjugates were purified from unreacted polymers and protein (Supporting Information) with a final SEC purification step (Figure 2B,C). Similar SEC elution times of each conjugate likely correlated with similar hydrodynamic radii. The dispersity of the conjugates, however, was not the same and corresponded with the dispersity of the attached polymer. The P(OEGMA^500^)_21_ conjugate peak (Figure 2B) was wider and therefore more disperse than the PDMA_61_ conjugate peak (Figure 2C). Fractions of the conjugate SEC peaks were selected for further characterization with SDS-PAGE (Figure 1D,E), which revealed that each sample contained some unmodified Gal3C[T243C] protein (band near 17 kDa marker on ladder). Concentrated fractions were pooled and used to measure binding affinity to an endogenous Gal3C ligand, LacNac, via changes in inherent tryptophan fluorescence upon ligand binding (Figure 1F) for W181 located adjacent to the ligand binding pocket, an established method for assessing Gal3C function^36^ (Figure 1F). Conjugation with either POEGMA or PDMA preserved protein function of Gal3C[T243C] with slightly increased binding affinities for LacNac over unmodified Gal3C[T243C] (Figure 2F).

### Assessment of Heterogeneity of Gal3C–Polymer Linker Chemistry

It was hypothesized that some of the conjugates were formed by disulfide bond coupling between C243 and polymer-thiols because the polymers featured subquantitative functionalization with the maleimide linker. Therefore, we wanted to assess if reducing conditions of SDS-PAGE enhanced the presence of the Gal3C[T243C] band by reducing disulfide bonds between polymer and protein. To interrogate this, individual fractions of each conjugate were selected, and SEC was performed pre- and postreduction with 10 mM DTT at 37 °C (Figure S8). The SEC traces prereduction (Figure S8B,C, black traces) revealed a small amount of Gal3C[T243C] in both conjugate samples. Postreduction (Figure S8 B,C, red dashed traces), the peak area of the conjugate decreased by 10–20% which indicated some disulfide bond linkages between the protein and polymer. Overall, this analysis suggested that conditions of the reducing SDS-PAGE gels presented herein may underestimate conjugate purity by 10–20%. Samples used for biophysical analyses were therefore prepared under nonreducing conditions. Minor heterogeneity in linker chemistry was not concerning based on previous work which suggests the linker has little effect on the galectin structure^33^ or function.^37^ HSQC spectra of Gal3C[T243C] conjugates did not show the presence of extensive peak doubling, as would be expected for heterogeneous samples containing a significant fraction of unconjugated protein (see below).

### Mapping Gal3C–Polymer Interactions via NMR Spectroscopy in Aqueous Solutions

To provide a residue-specific assessment of the impact of polymer conjugation on the Gal3C backbone structure and conformational dynamics at an atomic scale, we prepared uniformly ^15^N-labeled Gal3C[T243C] conjugated to PDMA_61_ or P(OEGMA^500^)_21_ and recorded two-dimensional heteronuclear single-quantum correlation (HSQC) spectra. The HSQC spectra were recorded at 30 °C with both polymer conjugates and were well-dispersed and consistent with properly folded proteins (Figures S9 and S10). Spectral resolution of both the unconjugated and conjugated proteins was also excellent, facilitating a careful comparison of the unconjugated and conjugated protein samples.

Because the chemical shifts of most signals for both conjugates were similar to those previously assigned for Gal3C,^38^ Gal3,^39^ and Gal3C[T243C]–PEG,^33^ we could transfer information about the assignments to spectra of the present conjugates with PDMA_61_ and P(OEGMA^500^)_21_. This allowed us to quantitatively compare the impact of polymer conjugation on the protein backbone structure and dynamics by reporting chemical shift perturbations and changes in signal line widths, respectively, upon polymer conjugation. This also facilitated a comparison of the impact of Gal3C conjugation with PDMA_61_ or P(OEGMA^500^)_21_ to earlier results of Gal3C conjugated with PEG to reveal possible relationships between polymer architecture and response of the protein to conjugation with each polymer.^33^

**Figure 3:**
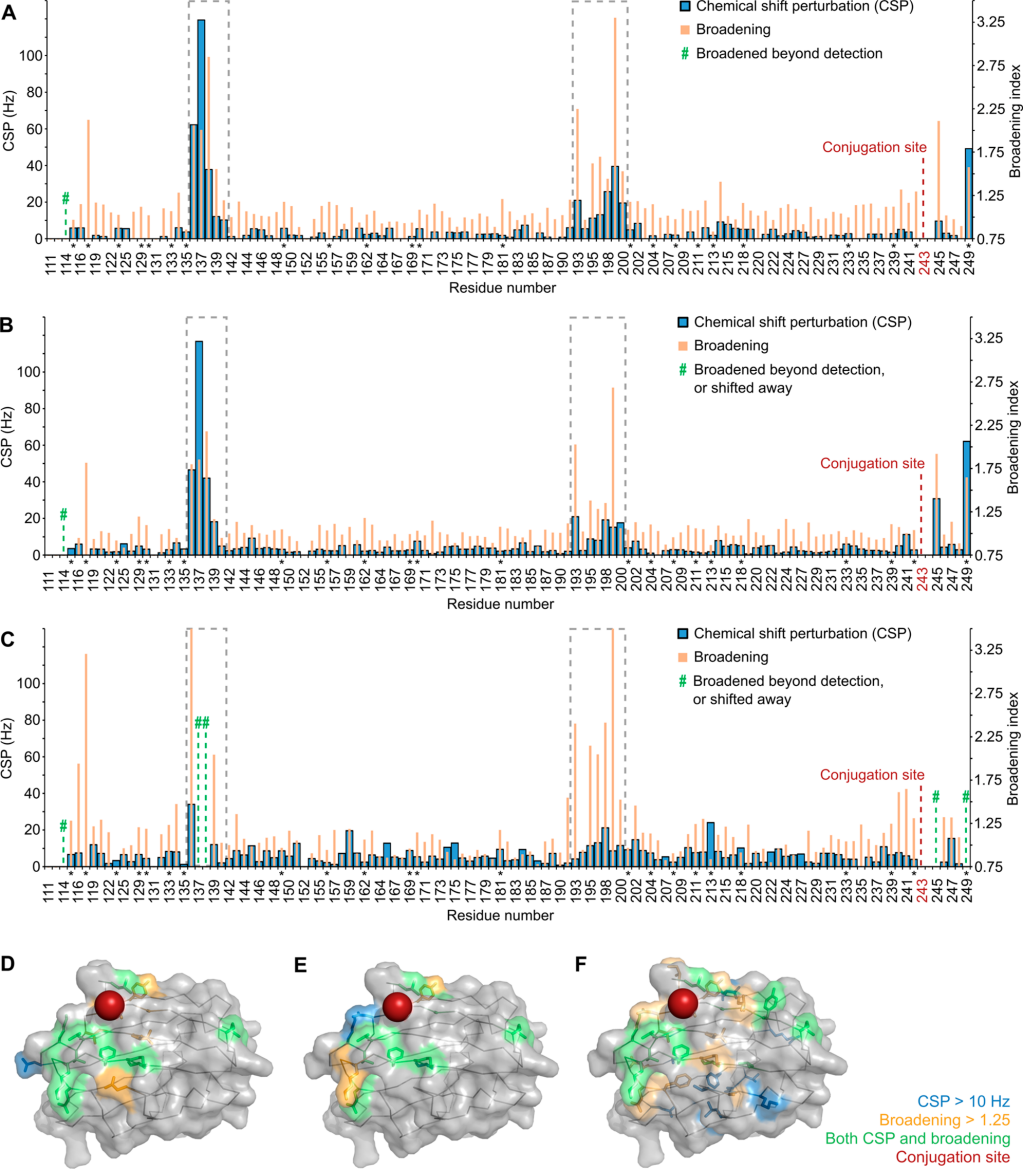
Effects of conjugation with 5.4 kDa PEG, PDMA_61_, and P(OEGMA^500^)_21_ on the backbone structure and dynamics of [U-^15^N]-Gal3C[T243C] as observed by chemical shift perturbations (CSPs) and line broadenings between [^15^N,^1^H]-HSQC NMR spectra of Gal3C[T243C] and conjugates. (A–C) Histograms reporting CSPs and line broadening of backbone amide signals for Gal3C[T243C] conjugated with (A) PEG, (B) PDMA_61_, and (C) P(OEGMA^500^)_21_. The “∗” indicates residues with ambiguous assignments. The green “#” indicates backbone amides broadened beyond detection or unassignable. The conjugation site, C243, is indicated by the red line. Areas of significant perturbation due to PEGylation at C243, as observed in a previous study of Gal3C[T243C], are outlined with gray dashed boxes on each plot. (D–F) Mapping chemical shift perturbations and line broadenings on the structure of Gal3C[T243C] for the conjugates with (D) PEG, (E) PDMA_61_, and (F) P(OEGMA^500^)_21_. Gal3C[T243C] (PDB ID 4R9A) is shown in space-filling representation with residues colored according to the lower right legend. Panels A and D are adapted with permission from ref (33). Copyright 2022 John Wiley & Sons.

Comparison of HSQC spectra of unmodified [U-^15^N]-Gal3C[T243C] and [U-^15^N]-Gal3C[T243C] conjugated to either PDMA_61_ or P(OEGMA^500^)_21_ revealed distinct impacts of each polymer on the protein, which are dependent on the polymer chemical scaffold. The conjugation of [U-^15^N]-Gal3C[T243C] with the linear polymer PDMA_61_ (Figure 3B) resulted in patterns of chemical shift perturbations and line broadening that were highly similar to those observed for Gal3C[T243C] conjugated with PEG (Figure 3A,D). The impact of conjugation of Gal3C with PDMA_61_ was further visualized by mapping residues showing either significant chemical shift perturbations (>10 Hz) or line broadening onto a structure of Gal3C (PDB ID 4R9A)^40^ (Figure 3). The largest impact was observed for residues local to the site of chemical conjugation, similar to Gal3C conjugates prepared with PEG,^33^ and similar patterns of effects on the Gal3C backbone were observed for PEG and PDMA_61_ (Figure 3). Though monomers for PDMA_61_ and PEG have different chemical structures (Figure 1), both polymers have linear architecture, indicating that similar polymer architectures result in similar patterns of protein–polymer interactions (Figure 3).

In contrast to the Gal3C[T243C] conjugate with PDMA_61_, we observed more significant line broadening for a wider range of residues in NMR data of the conjugate with P(OEGMA^500^)_21_ (Figure 3C and Figure S10). Broadening of signals in HSQC spectra of globular proteins has been attributed to fluctuations in the backbone conformation on the time scale of milliseconds to microseconds.^41^ Because we could not precisely delineate all potential contributions to line broadening, we focused on analyzing the patterns of protein–polymer interactions. For both conjugates with PDMA_61_ and P(OEGMA^500^)_21_, we observed significant line broadening and chemical shift differences for residues proximate to the site of conjugation. However, for the conjugate with P(OEGMA^500^)_21_, more significant line broadening was observed among the same set of residues, especially for residues at positions 135–140 and positions 192–200, which primarily comprise loops connecting two adjacent β-strands (Figure 3C,F). Interestingly, while conjugates prepared with PDMA and POEGMA shared similar sites of perturbations local to the conjugation site, Gal3C–POEGMA showed more extensive changes at residue positions farther from C243 (Figure 3C,F). The polymer backbones for PDMA or PEG are linear and typically adopt a more random-coil-like conformation in solution. Conversely, POEGMA, with its numerous branches or side chains, adopts an extended, comblike conformation in solution. POEGMA can transition between extended or collapsed conformations depending on factors such as the length and chemical structure of its side chains, salt concentrations, or temperature.^21^ The NMR data for Gal3C conjugated to P(OEGMA^500^)_21_ suggest a more extended conformation of the polymer in the conjugate, as changes in the HSQC NMR data were spread over a wider region of the protein surface (Figure 3), as opposed to perturbations that would be more confined locally to the site of chemical conjugation for a more compact polymer backbone. For both conjugates, line broadening was observed to be specific to individual residues, not uniformly observed for the entire protein, indicating that local changes in protein dynamics caused by conjugation were likely responsible, rather than significant changes in the diffusion of the conjugated proteins.

### Conjugation with PDMA and POEGMA Alters Gal3C Stability in a Polymer Length or Branch Length Dependent Manner

To interrogate the role of polymer length and chemical structure on the thermal stability of conjugated Gal3C[T243C], we recorded circular dichroism (CD) spectroscopy thermal melting assays with Gal3C[T243C] conjugated to each of the five polymers shown in Figure 1C, including PDMA_61_ and P(OEGMA^500^)_21_. To determine the effect of polymer conjugation on the unfolding pathway of Gal3C[T243C], discrete fractions of Gal3C[T243C]–polymer conjugates from SEC experiments (Table S1) were collected and subjected to continuous ramp thermal melting monitored by CD spectroscopy. Melting profiles were determined from full CD versus wavelength spectra recorded with an applied linear temperature ramp from 30 to 90 °C for each conjugate (Figure S11). Previously it was shown that conjugation of Gal3C[T243C] with PEG altered the unfolding pathway of Gal3C[T243C], resulting in a significantly higher thermal melting temperature (*T*_m_) via the formation of a polymer-dependent unfolding intermediate.^33^ It was thus of interest to investigate which factors were critical for improved protein stability, i.e., to what extent polymer architecture, degree of polymerization (*n*), or branch length (*m*) influenced the protein thermal unfolding profile.

**Figure 4:**
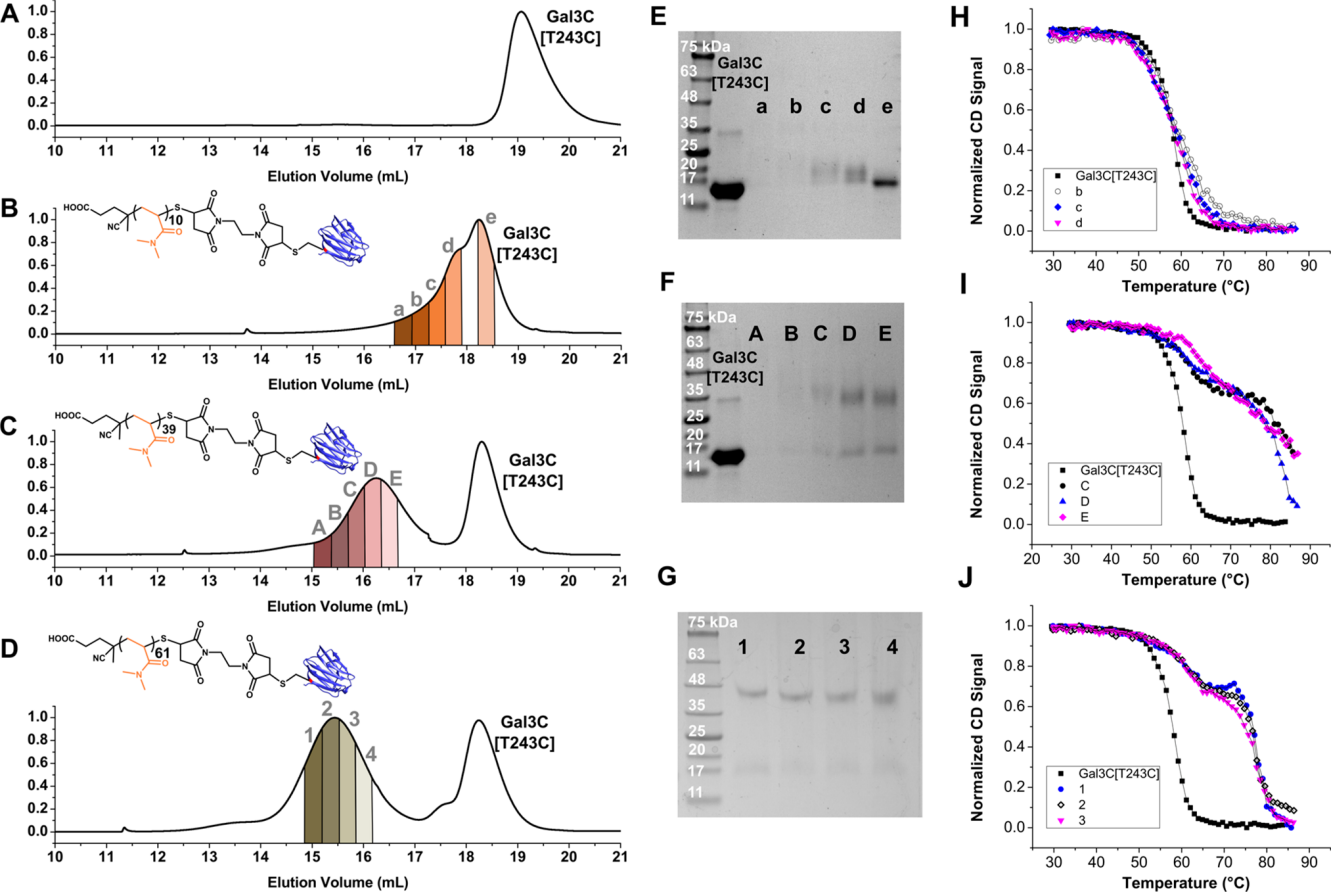
Preparation and thermal melting profiles of Gal3C[T243C]–PDMA_10,_ Gal3C[T243C]–PDMA_39_, and Gal3C[T243C]–PDMA_61_. (A) Reference chromatogram of unmodified Gal3C[T243C]. (B–D) SEC chromatograms of (B) Gal3C[T243C]–PDMA_10_, (C) Gal3C[T243C]–PDMA_39_, and (D) Gal3C[T243C]–PDMA_61_ with exact fractions selected for characterization labeled a–e, A–E, and 1–4, respectively. All fractions were 330 μL. (E–G) SDS-PAGE gels of select conjugate fractions for (E) Gal3C[T243C]–PDMA_10_, (F) Gal3C[T243C]–PDMA_39_, and (G) Gal3C[T243C]–PDMA_61_. (H–J) Thermal melting plots monitored by single-wavelength circular dichroism of (H) Gal3C[T243C]–PDMA_10_ fractions b–d, (I) Gal3C[T243C]–PDMA_39_ fractions C–E, and (J) Gal3C[T243C]–PDMA_61_ fractions 1–3. The thermal melting profile of unmodified Gal3C[T243C] is superimposed in panels H–J.

To delineate the role of polymer length on thermal unfolding of Gal3C conjugated to linear polymers, Gal3C was conjugated to PDMA of three different degrees of polymerization (*n* = 10, 39, and 61). Conjugated protein samples were evaluated via analytical SEC, SDS-PAGE, and CD-monitored thermal melting assays. Analytical SEC showed the presence of a peak for conjugated Gal3C that shifted left with increasing molecular weight, as expected (Figure 4A–D). For *n* values of 39 and 61, the peak for modified Gal3C could be baseline resolved from unmodified Gal3C. For *n* = 10, the modified and unmodified proteins were partially overlapped (Figure 4B). Discrete fractions of each conjugate were characterized by SDS-PAGE (Figure 4E–G) and thermal melting (Figure 4H–J and Table S1) and compared with unmodified Gal3C[T243C].

Figure 4H shows results of thermal melting of the PDMA_10_ conjugate fractions b–d (Figure 4B,E), which were selected to be enriched with conjugate rather than unmodified protein. Gal3C[T243C]–PDMA_10_ did not have observable intermediate formation, while Gal3C[T243C]–PDMA_39_ (Figure 4I) and Gal3C[T243C]–PDMA_61_ (Figure 4J) did. Additionally, the two higher molecular weight PDMA conjugates had similar thermal stabilities and unfolding profiles. The thermal unfolding data indicated the oligomer PDMA_10_ was not a large enough polymer to produce an intermediate, while PDMA_39_ and PDMA_61_ were long enough to significantly alter the Gal3C[T243C] thermal unfolding pathway. These results are reminiscent of earlier studies showing increased protein activity at higher temperatures with increased polymer chain lengths.^23,29^ From the thermal unfolding data, we concluded that a threshold polymer length for the linear PDMA was needed both for significant increased thermal stability and formation of a thermal unfolding intermediate.

Next, we used two comb-shaped POEGMA polymers to interrogate the effect of monomer branch length (*m*) on Gal3C thermal unfolding. We hypothesized that, for conjugates made with POEGMA, the polymer–polymer interactions of the methacrylate backbone and PEG oligo side chains may dominate over protein–polymer interactions, preventing the formation of thermal unfolding intermediates, especially for more hydrophobic, shorter branch length OEGMA^300^. Additionally, both POEGMA polymers exhibited a much lower *n* compared with intermediate forming polymers PDMA_61_ and PEG. To directly test this, conjugates of Gal3C[T243C] were prepared with P(OEGMA^500^)_21_ and P(OEGMA^300^)_20_. Degree of polymerization (*n*) was controlled to assess the role of branch length (*m*) in protein unfolding.

**Figure 5:**
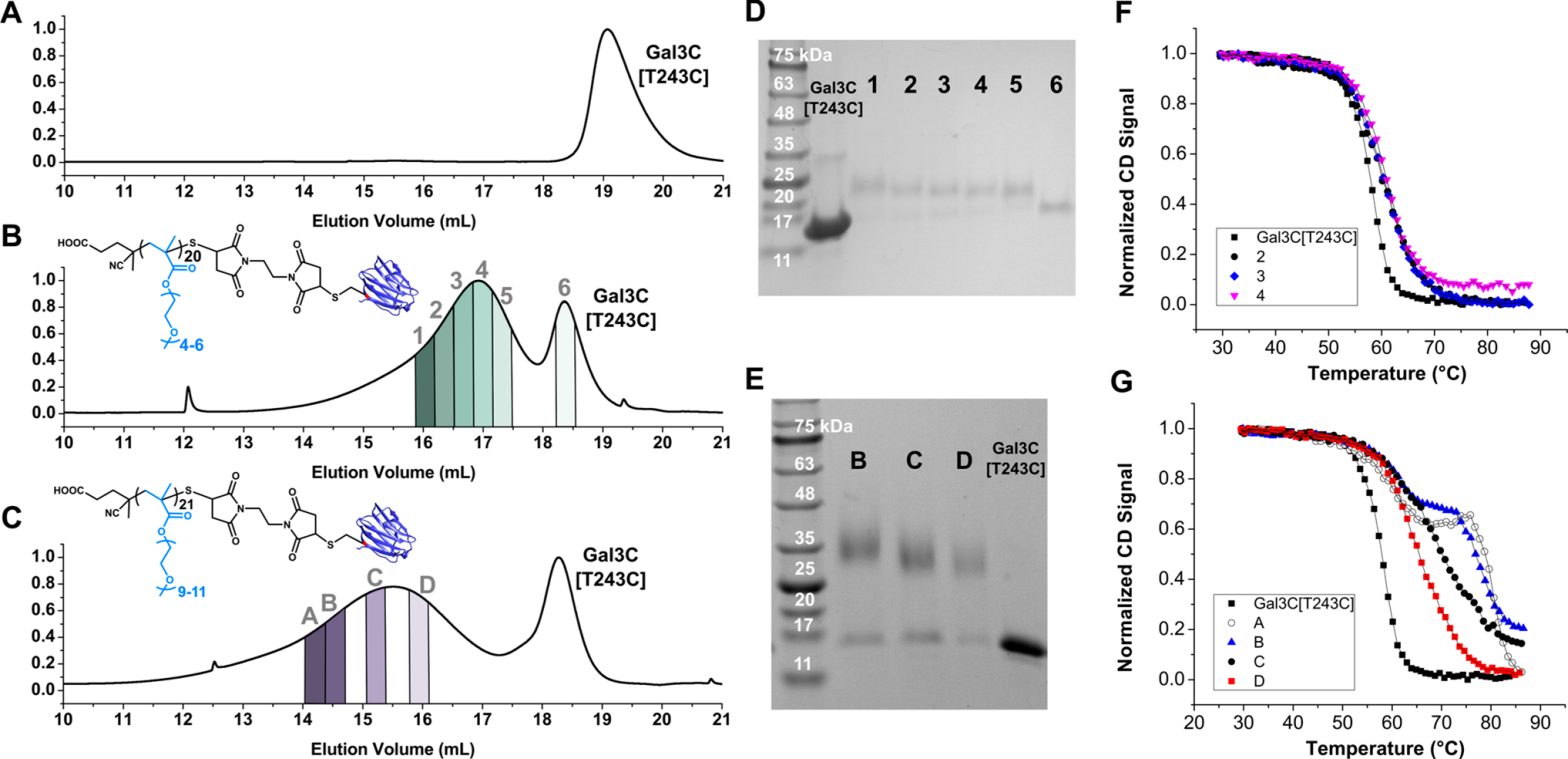
Preparation and thermal melting of Gal3C[T243C]–P(OEGMA^300^)_20_ and Gal3C[T243C]–P(OEGMA^500^)_21_. (A) Reference chromatogram of unmodified Gal3C[T243C]. (B) SEC purification of Gal3C[T243C]–P(OEGMA^300^)_20_ with exact fractions selected for characterization labeled 1–6. All fractions were 330 μL. (C) SEC purification of Gal3C[T243C]–P(OEGMA^500^)_21_ with exact fractions selected for characterization labeled A–D. (D and E) SDS-PAGE of select conjugate fractions for Gal3C[T243C]–P(OEGMA^300^)_20_ and Gal3C[T243C]–P(OEGMA^500^)_21_, respectively. The unmodified Gal3C[T243C] lane in (D) features both monomeric (17 kDa) and the occasionally observed dimer (34 kDa) in sample. (F) Single-wavelength thermal melting plots of Gal3C[T243C]–P(OEGMA^300^)_20_ fractions 2–4 as compared to unmodified Gal3C[T243C]. (G) Single-wavelength thermal melting plots of Gal3C[T243C]–P(OEGMA^500^)_21_ fractions A–D as compared to unmodified Gal3C[T243C].

SEC separations of Gal3C[T243C]–P(OEGMA^300^)_20_ and Gal3C[T243C]–P(OEGMA^500^)_21_ were mostly resolved from unconjugated protein in SEC separations (Figure 5B,C). Defined fractions of conjugated protein of varying molecular weight sizes were selected for SDS-PAGE analysis (Figure 5D,E) and thermal melting (Figure 5F,G). SDS-PAGE and SEC of the two conjugates showed consistent conjugate sizes as compared to unmodified Gal3C[T243C], which was observed as a contaminant in SDS-PAGE. The CD thermal melting assay revealed that all selected Gal3C[T243C]–P(OEGMA^300^)_20_ fractions did not form defined intermediate states, but had a slightly higher overall melting temperature as compared to unmodified Gal3C[T243C] (Figure 5F). Interestingly, thermal melting of Gal3C[T243C]–P(OEGMA^500^)_21_ revealed a striking increase in *T*_m_ for conjugates of higher molecular weight fractions (Figure 5C, fractions A and B). The same higher molecular weight fractions formed defined unfolding intermediate states, indicated by plateau regions from 65 to 80 °C (Figure 5G).

CD spectra of the intermediate state show that it is globally folded with mostly β sheet secondary structure (Figure S11). The global fold of the intermediate is likely similar to that of the conjugated protein at lower temperature, but a shift in the λ_min_ value from 222 to 218 nm over the intermediate formation temperature range indicates it is not identical. HSQC spectra of Gal3C[T243C]–P(OEGMA^500^)_21_ measured at 55 °C, the highest temperature we could safely operate the NMR probe, confirm the conjugated protein was folded (Figure S12). The transition from a two-state to a three-state melting curve was previously observed for Gal3C[T243C] conjugated to PEG_4.5k_ and attributed to sustained protein–polymer interactions at increasing temperatures.^33^

Lower molecular weight fractions of Gal3C[T243C]–P(OEGMA^500^)_21_ (C and D) exhibited a qualitatively higher *T*_m_ than the unmodified protein but lacked the plateauing indicative of a defined intermediate (Figure 5G, black circles and red squares). Comparison of thermal unfolding results from the two POEGMA-conjugated protein samples suggested the hydrophobic nature of P(OEGMA^300^)_20_ (short branch length, *m* = 4–6) may have reduced protein–polymer interactions required to redirect the protein thermal unfolding pathway. For the less hydrophobic and higher molecular weight P(OEGMA^500^)_21_, a greater number of protein–polymer interactions may lead to the formation of a clear thermal unfolding intermediate state.

Comparing observations of thermal unfolding of Gal3C[T243C] conjugated to the linear PDMA and to the nonlinear POEGMA polymers enabled us to investigate potential correlations between polymer architecture and the thermal unfolding behavior of conjugated proteins. For both PDMA and POEGMA, a minimal polymer length was needed to form the thermal unfolding intermediate state. Interestingly, the intermediate states observed for both longer chain PDMA and POEGMA conjugates formed over similar temperature ranges. Intermediate states for both the PDMA and POEGMA conjugated protein and exhibited similar spectral signatures in the full CD versus wavelength melting spectra, as observed by the λ minimum shifting from 222 to 218 nm for the unfolding intermediate state (Figure S10C,F). This suggested both polymers lead to similar intermediate global structures which are distinct from the native global structure. Because polymers can form amphiphilic interactions with the protein surface,^33,42^ it is conceivable that any neutral polymer above a certain threshold length may be able to form a sufficient amount of interactions with the protein to redirect the unfolding pathway, which may explain similar intermediate formation pathways shared among Gal3C[T243C] conjugated to P(OEGMA^500^)_21_, PDMA_61_, or PEG.

## Conclusion

The findings presented in this study demonstrate that Gal3C conjugated with either PDMA or POEGMA, two PEG-alternative polymers, achieves comparable improvements in protein thermal stability as when Gal3C is conjugated with PEG. This indicates that both linear and nonlinear polymer chemical architectures can effectively improve protein stability. NMR analysis of the Gal3C–PDMA conjugate showed patterns of chemical shifts and line broadenings similar to those observed for the Gal3C–PEG conjugate (Figure 3). The NMR spectra of the Gal3C–POEGMA conjugate showed more significant differences that extended farther from the site of chemical conjugation, suggesting the polymer adopted a more extended conformation that covered more of the protein surface (Figure 3). It is interesting to note that residues observed to be perturbed in the NMR spectra of Gal3C–PDMA were also observed to be perturbed in spectra of Gal3C–POEGMA, suggesting that the protein–polymer interactions underlying improved protein stability may be conserved for different polymers. The conformation of the thermal unfolding intermediates for PDMA and POEGMA conjugates also likely had similar global structures, as reflected by similar CD spectra.

Systematic comparison of the effect of polymer length and branching provide data that could aid in the development of criteria for the rational design of protein–polymer conjugates. Gal3C–PDMA conjugates with higher degrees of polymerization demonstrated thermal unfolding intermediates, while for the shortest polymer only a marginal increase in the unfolding temperature was observed (Figure 4). The thermal unfolding pathways of Gal3C[T243C] conjugates prepared with POEGMA also showed a clear dependence on degree of polymerization and branch length. For conjugates prepared with PDMA, we hypothesized a longer polymer was required to facilitate protein–polymer interactions present during the unfolding process, or longer polymers are required to act as an effective molecular “shield” preventing protein–protein aggregation. For conjugates prepared with POEGMA, we hypothesized that the more hydrophobic nature of short-branched POEGMA prevented extensive protein–polymer interactions required for stabilization and formation of the unfolding intermediate, suggesting a threshold polymer length or branch length is needed for redirection of the unfolding pathway.

## Experimental Section

Five total PDMA and POEGMA polymers were synthesized using RAFT polymerization and end-group functionalization prior to conjugation to Gal3C[T243C]. Aminonolysis was used to cleave trithiocarbonate groups and reveal polymer thiols. These were then reacted asymmetrically with a bismaleimide linker molecule through thiol-Michael addition. Both end-group-functionalization steps were performed in buffer and rapidly purified using a PD-10 desalting column monitored by measuring UV–vis absorption of elution fractions. Gal3C[T243C] was expressed and purified according to earlier reports.^33^ Gal3C[T243C] conjugates were generated with a grafting-to method using thiol-Michael addition between the polymer-maleimide and the protein at position C243. Unreacted Gal3C[T243C] and excess polymer were removed using lactose-affinity chromatography followed by size exclusion chromatography.

Individual fractions of the conjugate SEC peak were analyzed using SDS-PAGE and thermal melting monitored by variable-temperature CD spectroscopy recorded with an Applied Photophysics Chirascan spectrophotometer operating with Chirascan v4.7.0. CD spectra were acquired from 200 to 280 nm, and variable temperature data were recorded with a ramp from 30 to 90 °C. For [^15^N,^1^H]-HSQC NMR experiments, purified fractions of conjugated Gal3C[T243C] were pooled and concentrated to 20–40 μM in 20 mM sodium phosphate pH 6.9, 30 mM NaCl, 9.5% ^2^H_2_O. NMR spectra were recorded with a Bruker Avance III 800 MHz spectrometer equipped with a 5 mm TXI cryoprobe. Equilibrium ligand binding experiments were recorded using intrinsic tryptophan fluorescence spectroscopy. Full experimental methods are provided in the Supporting Information.

## Supporting Information Available

Detailed experimental procedures and methodologies, data analysis details, and additional data; materials and methods, polymer synthesis, NMR data of synthesis intermediates and products, protein production details, grafting-to conjugation details, variable temperature CD spectra, fluorescence equilibrium binding data and analysis, NMR spectra of conjugates, references (PDF)
Transparent Peer Review report available (PDF)

## Abbreviations

SEC: size exclusion chromatography
SDS-PAGE: sodium dodecyl sulfate polyacrylamide gel electrophoresis
PEG: poly(ethylene glycol)
POEGMA: poly(oligoethylene glycol methacrylate)
PDMA: poly(dimethylacrylamide)
Gal3C: carbohydrate recognition domain of human galectin protein Gal3
NMR: nuclear magnetic resonance
