# Supplementary material for: Conserved Protein–Polymer Interactions across Structurally Diverse Polymers Underlie Alterations to Protein Thermal Unfolding
Source: ACS Cent Sci. 2023 Mar 14;9(4):685–95. doi: 10.1021/acscentsci.2c01522 (PMC10146661; doi:10.1021/acscentsci.2c01522)
Supplement: Supplementary file 1 — oc2c01522_si_001.pdf [file oc2c01522_si_001.pdf]

Supplementary Information

Conserved Protein-Polymer Interactions Across Structurally Diverse Polymers Underly Alterations to Protein Thermal Unfolding

Amanda Pritzlaff[1], Guillaume Ferré[1,†], Elia Dargassies, Crystal O. Williams, Daniel D. Gonzalez, Matthew T. Eddy[1,*]

[1]*Department of Chemistry, University of Florida, 126 Sisler Hall, Gainesville, FL 32611, USA*

*To whom correspondence should be addressed; email: matthew.eddy@ufl.edu

Contents

SI Experimental Section 2

Materials 2

Polymer Synthesis 2

Synthesis of (1) bis-maleimide [1,1'-(ethane-1,2-diyl)bis(1*H*-pyrrole-2,5-dione)]: 2

NMR of Organic Molecules 3

Protein [^{15}N,^{1}H]-HSQC NMR: .... **Error! Bookmark not defined.**

Gel Permeation Chromatography: .... **Error! Bookmark not defined.**

Polymer End Group Functionalization 3

Grafting-to Conjugation and Purification 3

Protein Production 4

CD Spectroscopy Thermal Melting 5

Fluorescence Equilibrium Binding Assay 6

Supplemental Figures, Scheme, and Table 7

References 21

SI Experimental Section

Materials

Poly(ethylene glycol) mono methyl ether (mPEG, Mn = 5k g/mol) was purchased from Sigma Aldrich. Mesyl chloride (Ms, >99%) was purchased from TCI America. Dimethylformamide (DMF, 99.8%), dichloromethane (DCM), chloroform ($CHCl_3$) and triethyl amine (TEA, 99%), anhydrous sodium acetate (NaAc) and 29% NH_4OH were purchased from Fisher Chemical. Sodium phosphate monobasic monohydrate ($NaH_2PO_4 \cdot H_2O$), sodium phosphate dibasic anhydrous (Na_2HPO_4) and sodium chloride were purchased from Fisher Chemical. Dithiothreitol (DTT, >99%) was purchased from GoldBio. Hydrazine monohydrate (65% N_2H_4, 98% reagent grade) was from Sigma. Ethylene diamine (99%), maleic anhydride (99%), and acetic anhydride (99%) were purchased from Oakwood Chemical. The 4-(((2-Carboxyethyl)thio)carbonothioyl)thio)-4-cyanopentanoic acid) CTA (95%) was purchased from Boron Molecular. N,N-dimethylacrylamide (DMA, 99%) was from Sigma-Aldrich (stabilized). Poly(ethylene glycol) methacrylate (PEGMA) was from Sigma-Aldrich. DMA and PEGMA were filtered over basic alumina plugs before use. Azobisisobutyronitrile (AIBN, 98%) was purchased from Sigma Aldrich and recrystallized from methanol before use.

Polymer Synthesis

Polymer functionalization is shown in Scheme S1. Before polymerization, DMA and OEGMA monomers and dioxane solvent were purified by passing over a plug of basic alumina. The solvent volume was selected to dissolve monomers at a concentration of 2M. Solid CTA (4-(((2-carboxyethyl)thio)carbonothioyl)thio)-4-cyanopentanoic acid), 6×10^{-4} mol, 200-300 mg, 1 equiv.), recrystallized AIBN initiator (6×10^{-5} mol, 11 mg, 0.10 equiv.), DMF internal standard (300 uL), and monomer were dissolved in dioxane in a 50 mL Schlenk flask. Flasks were sealed and purged for 30 minutes with N_2 at room temperature before initiating polymerization in a 70 °C oil bath. Polymerization was monitored with GPC and ^{1}H NMR spectroscopy. When target conversion was reached, the flask was removed from heat and exposed to oxygen to quench the reaction. Polymers were isolated by 3X precipitation in 10 volume equivalents of cold ether. The solid (PDMA) or oil (POEGMA) polymers were isolated and dried under high vacuum overnight before final characterization. The specific monomer amounts, polymerization times, monomer conversion %, and yields are listed for each of the 5 polymers as follows: $POEGMA^{500}_{21}$ (0.0225 mol, 10.5 mL, 19.7 equiv. / 180 min / 55% / 6.03 g). $POEGMA^{300}_{20}$ (0.0224 mol, 6.45 mL, 19.7 equiv. / 160 min / 54% / 2.50 g). $PDMA_{10}$ (0.012 mol, 1.21 mL, 25 equiv. / 185 min / 32% / 0.180 g). $PDMA_{39}$ (0.026 mol, 2.68 mL, 40 equiv. / 165 min / 93% / 2.09 g). $PDMA_{62}$ (0.062 mol, 6.40 mL, 95 equiv / 115 min / 65% / 3.00 g).

Synthesis of (1) bis-maleimide [1,1'-(ethane-1,2-diyl)bis(1*H*-pyrrole-2,5-dione)]:

To generate the bis-amide intermediate, a literature procedure from Sava, M. *et al.* was adapted.[1] Ethylene diamine (1 equiv., 10 mmol, 0.60 g, 0.67 mL) in 25 mL of acetone was added dropwise to a solution of maleic anhydride (2.5 equiv., 28 mmol, 2.78 g) in 20 mL of acetone in a 100 mL flask. White precipitate was observed immediately upon addition of the amine. After 1.5 h, the reaction was heated to 35 °C and stirred for 30 more minutes. The flask was then cooled to room temperature and the solid was filtered out and dried under vacuum. The filtrate was stirred for 2 more days and more solid was collected. The bis-amide intermediate was confirmed by ^{1}H NMR and the solid used as-is in the next reaction.

To ring close the product and generate (1), a procedure from Cava, M. P., *et al.* was adapted.[2] The crude bis-maleamic acid (1 equiv., 7.93 mmol, 2.03 g) was dissolved in 10 mL of acetic anhydride in a 25 mL round bottom flask. Sodium acetate (2.2 equiv., 17.4 mmol, 1.43 g) was added to the flask and was sparingly soluble. The flask was then sealed, purged, and stirred at 100 °C for 35 minutes until one major product spot was visible on TLC. The reaction was quenched with 50 mL of cold water and an opaque, dull precipitate was observed and filtered off for further purification. The aqueous solution was then extracted 3x with ethyl acetate and the ethyl acetate layer and the previously precipitated solid were combined and purified via silica chromatography with 1L of 1:1 hexane: ethyl acetate. Final recovery of (1) was 720 mg / 3 mmol / 30% yield. The ^{1}H NMR of (1) is shown in Figure S6.

NMR Spectroscopy of Small Molecules and Polymers

All ^{1}H NMR samples were analyzed using a 500 MHz Varian or 400 MHz Bruker instrument and prepared in D_2O or $CDCl_3$ with approximate concentrations of: 5 mg/mL for all small molecules or 10-15 mg/mL for all polymers.

Polymer End Group Functionalization

Step 1: Aminolysis. The procedure from Shen *et al.* [3], was followed with some changes to carry out the CTA cleavage in PBS buffer. Generally, polymers were dissolved at a concentration of 100 mg/mL in 1-2 mL of 1XPBS. Hydrazine monohydrate (5 equiv.) was added to the reaction and it was immediately sealed and purged with N_2 before stirring. The reactions were monitored visually for disappearance of yellow CTA color and via UV-Vis with a Nanodrop spectrophotometer at 315 nm. In PBS, quantitative trithiocarbonate cleavage (Figure S5) took 1-2 hours. The reaction was then cleaned up via desalting using manufacturer protocols for either an AKTA Pure system equipped with a Cytiva 5 mL HiTrap desalting column (1.5 mL max loading volume) or a Cytiva PD-10 gravity desalting column with Sephadex G25 resin (1.75 – 2.5 mL loading volume). Collected fractions were monitored via UV-vis. A 90% recovery of polymer was assumed for each desalted sample to calculate final polymer yield.

Step 2: Thiol-Michael addition of bis-maleimide (1). Immediately following aminolysis cleanup, 1-2 mL portions of the polymer-SH in buffer were reacted with bis-maleimide (1) (pre-dissolved in DMF for final reaction concentration of 25% DMF) and triethylamine (1 equiv.). All solutions were purged with N_2 before and immediately after addition of reagents into sealed 10 mL flasks. The total reaction time was 4h at room temperature. Samples were again desalted to purify while tracking absorbance of fractions at 280 nm. Fractions were pooled and the final concentration of polymer was calculated assuming 90% recovery, per manufacturer standards. Maleimide/DMF could be seen eluting after the polymer via UV-vis measurement of fractions at 280 nm. To quantify conversion, aliquots of the polymer solution were dried down to solid buffer salts and polymer and redissolved in $CDCl_3$. The conversion was calculated using POEGMA ^{1}H NMR spectra. Integration of the maleimide 6.7 ppm peaks against the sharp 3.35 ppm methyl peak resulted in 12 – 21 % conversion. Stocks were either used fresh for conjugation or flash frozen.

Grafting-to Conjugation and Purification

Conjugates were prepared with stock solutions of polymer targeting 10 equiv. of polymer:protein. Aliquots of polymer stock of 0.3-0.5 mL were used to obtain the target equivalents. A range of 5-10 mg of Gal3C[T243C] (>100 μM) was reacted with the polymer stock in 1XPBS buffer, at room temperature, while stirring gently for 4h.

Lactose affinity chromatography was used to remove unreacted polymer and any unfolded protein from the reaction. The reaction was first quenched with 10 mM DTT and bound to lactose-agarose resin (10 mg protein: 1 mL resin) by rotating at 4 °C for 2 h. The mixture was then placed in a mini-column, washed with 10CV of cold PBS (up to 3 mL), and eluted with 30 mM lactose in PBS in single 0.25-0.5 mL aliquots-each following a 15-minute incubation time where the column was just capped.

Size exclusion chromatography (SEC) was performed as a final purification step using an AKTA system with a Superdex 200 Increase 10/300 GL column (0.5 mL / min, inject at 0.05 CV, isocratic elution for 1.1 CV). Protein-polymer conjugates were analyzed using SDS-PAGE with a reducing tris tricine buffer system. Conjugate fractions were collected in sequential 0.330 mL volumes from 0.55 – 0.86 CV for screening.

For the control Gal3C[T243C] reaction with 10 equiv. of PEG-mal (Figure S7), 8.1 mg (10 equiv.) of PEG was reacted with 1.29 mg (1 equiv.) of protein at room temperature in 1.5 mL PBS. At the target time points, 0.5 mL aliquots were selected for SEC and SDS-PAGE, without further purification of the conjugate (from excess PEG-mal) performed. The procedure for synthesis of PEG-mal from purchased mono-methoxy PEG was reported previously.[4]

Protein Production

The plasmid containing Gal3C[T243C] with a 6 X C-terminal His-tag was transformed into *E. coli* Origami B(DE3) competent cells. Protein expression was carried out using a previously reported protocol.[4] Unlabeled Gal3C[T243C] was expressed in 2 x TY media. [u-^{15}N]-Gal3C[T243C] was expressed in M9 minimal media containing 1 g/L ^{15}N ammonium chloride (Cambridge Isotope Labs).

Gal3C[T243C] purification followed previously reported procedures.[4] In brief, thawed cell pellets were resuspended in cold PBS, 10 mM DTT and protease inhibitor and lysed three passages through a cell disruptor (Pressure Biosciences) at 25 kpsi. The lysate was clarified by centrifugation, filtered with 0.45 µM GF+PES syringe filters, and mixed with cobalt IMAC resin at the ratio of 0.5 mL resin per 2 L of original culture volume in conical tubes. The filtered lysate and resin were rotated at 4 °C for 20 – 30 minutes then washed with 1XPBS (50 mL), 1XPBS and 500 mM NaCl (50 mL), and 1XPBS and 5 mM imidazole (30 mL). The protein was eluted with 5 mL of 1XPBS and 75 mM imidazole after first allowing the buffer to enter the resin bed and incubated for 15 minutes. 0.5 mL fractions were collected and highest concentration fractions pooled.

Gel Permeation Chromatography:

Number-averaged molecular weights (M_n) and dispersity were obtained by performing GPC in N,N-dimethylacetamide (DMAC) with 50 mM LiCl at 50 °C with a flow rate of 1.0 mL/min and using multi-angle light scattering detection (Agilent Infinity II isocratic pump, degasser and autosampler and ViscoGel I-series 5 µm guard column, Malvern I-MBLMW and IMBHMW 3078 columns with an exclusion limit of 20,000 g/mol and 1.0 x 10^7 g/mol, respectively). Conventional calibration with poly(methyl methacrylate) standards was employed for POEGMA samples. The detection sources were a Wyatt Optilab T-rEX refractive index detector operating at 658 nm and a Wyatt miniDAWN Treos light scattering detector operating at 659 nm. Molecular weights and molecular weight distributions were calculated using the Wyatt ASTRA software

Protein NMR Sample Preparation, Data Acquisition and Analysis

NMR sample preparation, data acquisition and processing followed previously described protocols to be consistent with earlier work.[4] NMR samples were concentrated to 20 µM for [u-^{15}N]-Gal3C[T243C]-P(OEGMA^{500})$_{21}$ and 40 µM for [u-^{15}N]-Gal3C[T243C]-PDMA$_{61}$. The POEGMA conjugated NMR sample was prepared by pooling fractions from the top of the conjugate peak ranging from 15.05 - 15.71 mL (Fraction C and immediately adjacent fractions), as shown in Figure 5. The PDMA conjugated NMR sample was prepared by pooling fractions from 15.19 - 15.85 mL (Fractions 2 and 3 in Figure 4). NMR samples were exchanged into NMR buffer (20 mM sodium phosphate pH 6.9, 30 mM NaCl) via a final SEC purification step and ^2H_2O was added to the final NMR sample to a final 9.5% v/v ratio.

NMR data were recorded with a Bruker Avance III spectrometer operating at 800 MHz, running Topspin version 3.6.3 and equipped with a 5 mm TXI cryoprobe. The temperature was calibrated using a standard sample of 4% methanol in d_4-MeOH. 2D [^{15}N,^{1}H]-HSQC spectra were recorded using a gradient sensitivity-enhanced pulse sequence (hsqcetf3gpsi) with 2048 points in the direct dimension, 180 points in the indirect dimension and 736 or 400 scans for [u-^{15}N]-Gal3C[T243C]-P(OEGMA^{500})$_{21}$ and [u-^{15}N]-Gal3C[T243C]-PDMA$_{61}$, respectively.

NMR spectra were processed with TopSpin 3.2 and analyzed with NMRFAM-SPARKY version 1.470. Prior to Fourier transformation, the data matrices were zero filled to 1024 (t1) x 4096 (t2) complex points and multiplied by 6.0 Hz gaussian and cosine window functions applied to the direct and indirect dimensions, respectively. Chemical shift perturbations and broadening indices were calculated by comparing conjugate spectra to [u-^{15}N]-Gal3C[T243C]. Chemical shift perturbations of the backbone amide signals were quantified using the following equation

$$\mathrm{CSP\ (conjugate)} = \sqrt{\left(\omega_{\mathrm{conjugate}} - \omega_{\mathrm{no\ conjugate}}\right)_{\mathrm{H}}^{2} + \left(\omega_{\mathrm{conjugate}} - \omega_{\mathrm{no\ conjugate}}\right)_{\mathrm{N}}^{2}}$$

where $\omega_{\mathrm{conjugate}}$ and $\omega_{\mathrm{no\ conjugate}}$ are the resonance frequencies of a given amino acid backbone amide for the conjugated and unconjugated protein, respectively, and where H and N written in subscripts outside of the parentheses indicate the amide chemical shifts for the ^{1}H and ^{15}N dimensions, respectively.

The extent of line broadening of NMR signals due to conjugation ($\mathrm{Broadening\ (conjugate)}$) was quantified using the following equation

$$\mathrm{Broadening\ (conjugate)} = \frac{\left(\frac{\mathrm{I_{residue}}}{\mathrm{I_{max}}}\right)_{\mathrm{no\ conjugate}}}{\left(\frac{\mathrm{I_{residue}}}{\mathrm{I_{max}}}\right)_{\mathrm{conjugate}}}$$

where $\mathrm{I_{residue}}$ is the signal intensity of a given amino acid backbone amide and $\mathrm{I_{max}}$ is the most intense signal in the spectrum and where $\mathrm{no\ conjugate}$ and $\mathrm{conjugate}$ outside of the parentheses designate the unconjugated and conjugated protein, respectively.

Circular Dichorism (CD) Spectroscopy Thermal Melting and Analysis

CD spectroscopic data were recorded with an Applied Photophysics Chirascan spectrophotometer equipped with a Quantum Northwestern Peltier temperature control device and operating with Chirascan v4.7.0 and Pro-Data Viewer software, following procedures used in our previous study.[4] Spectra were background subtracted against the sample buffer. For thermal

unfolding experiments, a linear thermal ramp was applied from 30 °C to 90 °C at 1 °C per minute with samples concentrated to 10 μM in PBS. All spectra were measured in triplicate, and data were normalized to account for slight differences in protein concentration.

Full wavelength spectra for each construct (Figure S10) were used to calculate a wavelength of greatest change (222 or 224 nm) to create single wavelength CD vs. temperature plots (Figures 4 and 5). The single wavelength CD signal vs. temperature plot of Gal3C[T243C] used data acquired at 220 nm, which was the wavelength of greatest change for the unmodified protein. To calculate melting temperatures, the normalized data were fit to a Boltzmann sigmoidal function in Origin v8.5. For the data fitting, no parameters were fixed, and the 'x' parameter defined as the center of the data was interpreted as the melting temperature (T_m).

Fluorescence Equilibrium Binding Assay

Equilibrium binding data acquisition and fitting were done according to previous work.[4] Intrinsic tryptophan fluorescence was monitored using a Cary Eclipse Fluorescence Spectrophotometer (Agilent) operating with Cary Eclipse WinFLR software version 1.2 in 'Scan Mode' with an excitation wavelength of 280 nm, acquisition range from 290 nm to 440 nm and photomultiplier tube sensitivity set to 'medium'. Samples of Gal3C[T243C] conjugates were prepared by pooling SEC fractions, as was done to prepare NMR samples (above), and diluting to 5-6 μM in PBS. Samples were titrated with the N-acetyllactosamine (LacNac) ligand until equilibrium binding conditions were established of at least 10 times the theoretical K_D, recording at least 20 data points per titration.

"Hill 1 equation" in Origin v8.5 software, describing reversible binding of a protein to a ligand, was used to fit the normalized equilibrium binding data. Fixed parameters included the Start and End values, which were set to 1 and 0, respectively. Replicated experiments with a fit error of ≤ 30% were used to the calculate the average K_D ± standard deviation with a total of 3 independent replicated experiments

Supplemental Figures, Scheme, and Table

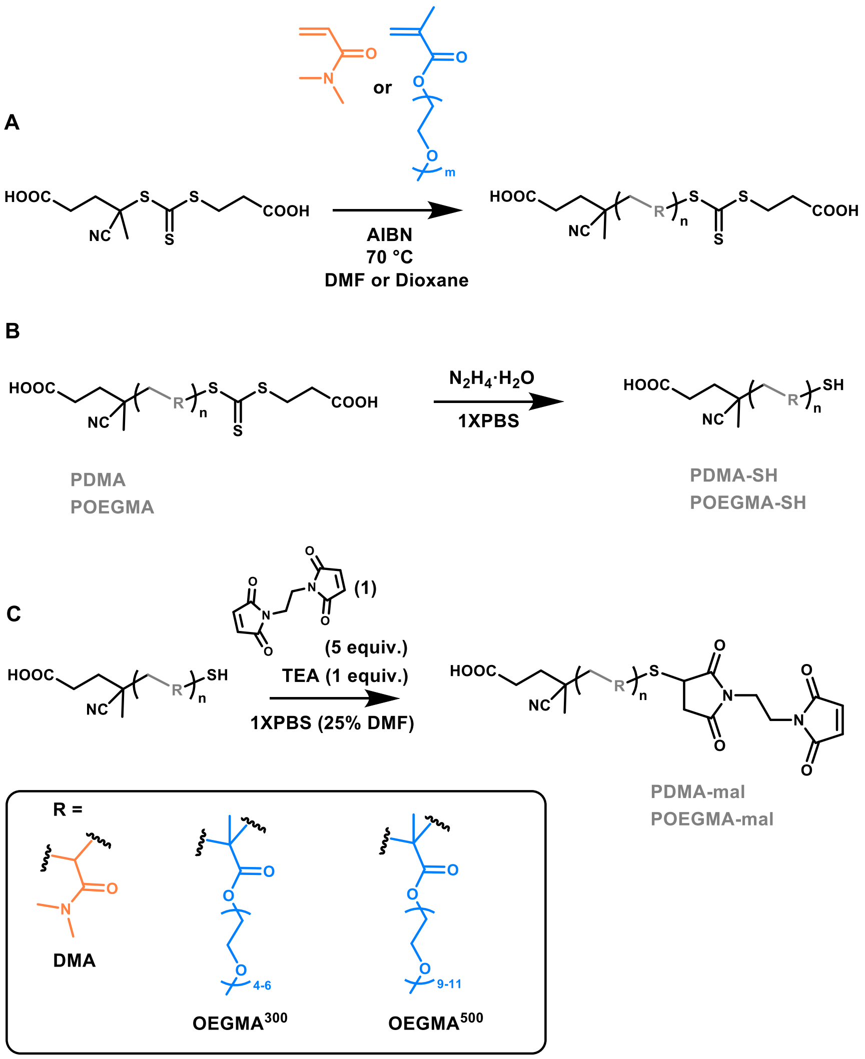

Scheme S1. Two-step scheme for functionalization of trithiocarbonate end-group polymers with bis-maleimide linker (1). Each step was followed by desalting in PBS.

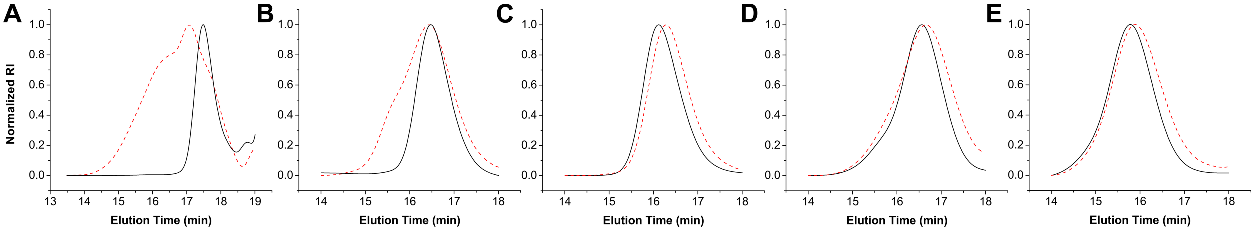

Figure S1. Size exclusion chromatograms of polymers obtained using gel permeation chromatography before (solid black lines) and after (red dashed lines) end-group functionalization with bismaleimide linker. (A) $PDMA_{10}$, (B) $PDMA_{39}$, and (C) $PDMA_{61}$; (D) $P(OEGMA^{500})_{21}$ and (E) $P(OEGMA^{300})_{20}$ in DMAC solvent.

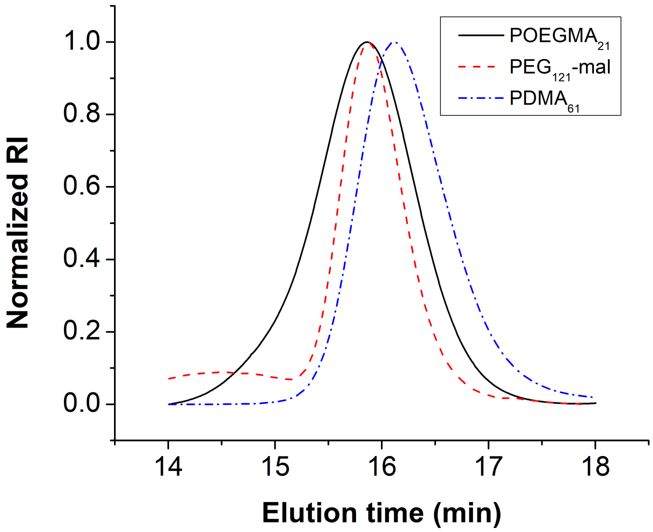

Figure S2. Superimposed size exclusion chromatograms of $P(OEGMA^{500})_{21}$ (solid black line), $PDMA_{61}$ (blue dash-dotted line) and reference 5,000 g/mol PEG_{121}-maleimide (red dashed line) in DMAC solvent.

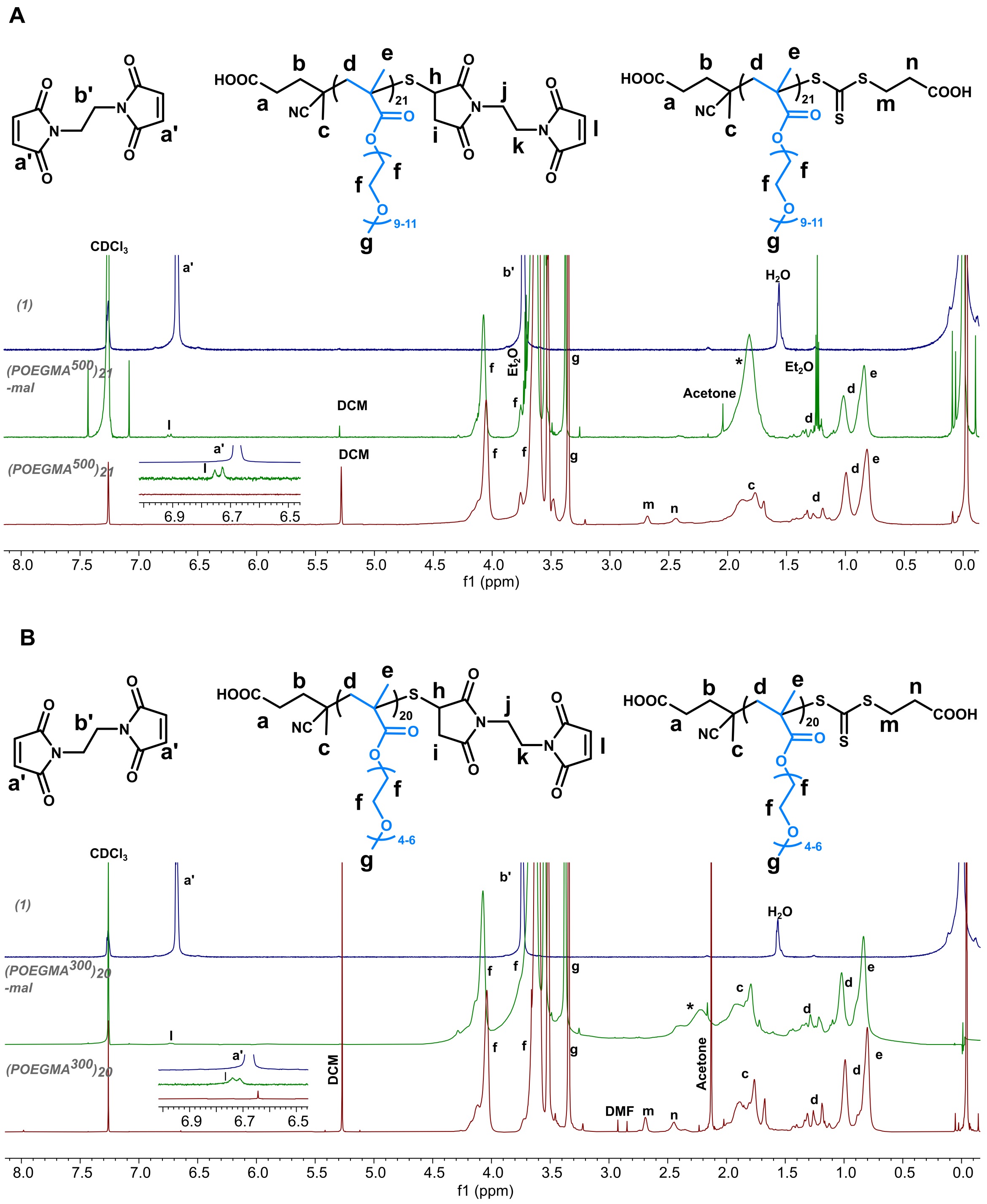

Figure S3. ^{1}H NMR spectra of POEGMA polymers before and after functionalization with bis-maleimide (1), (**A**) P(OEGMA^{500})$_{21}$, and (**B**) P(OEGMA^{300})$_{20}$. Bis-maleimide linker (1) is provided as the top spectrum in A and B in blue. End-group functionalized polymers are shown in the middle spectra in green. Unmodified polymers are shown in the bottom red spectra. Inset spectra show the maleimide region of the spectra from δ 6.5 – 6.95 ppm. Broad peaks labeled with an asterisk (*) in the green spectra in A and B are impurity peaks related to isolating polymer from buffer prior to dissolving in NMR solvent. Spectra were recorded on a 500 MHz Varian instrument in $CDCl_3$ (see methods).

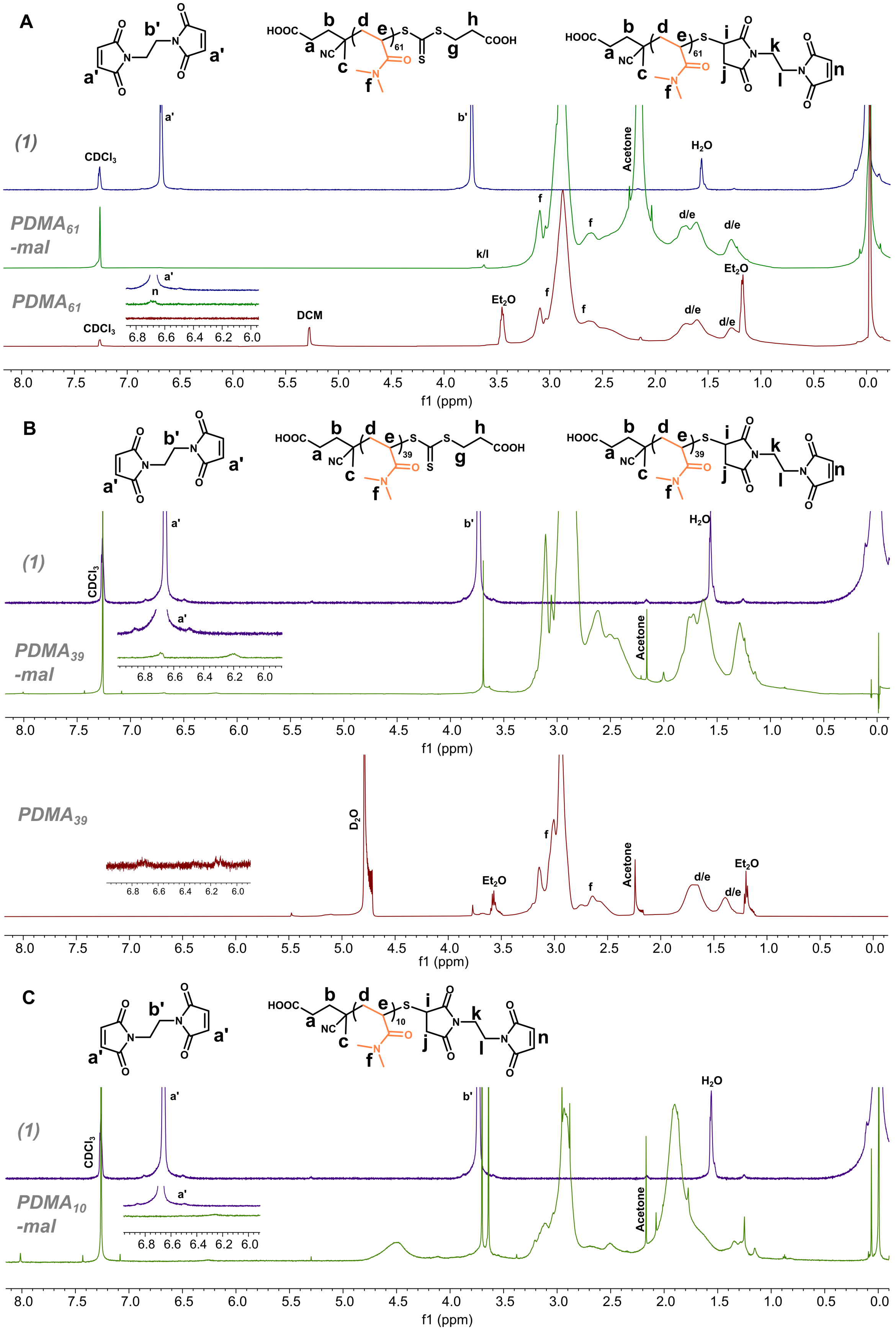
A
B
C
(1)
PDMA61 -mal
PDMA61
PDMA39 -mal
PDMA39
PDMA10 -mal
CDCl3
DCM
Et2O
Acetone
H2O
D2O
f1 (ppm)

Figure S4. ^{1}H NMR spectra of PDMA polymers before and after functionalization with bis-maleimide (1), (**A**) $PDMA_{61}$, (**B**) $PDMA_{39}$, and (**C**)$PDMA_{10}$. Bis-maleimide linker (1) is shown as the top spectrum in blue. End-group functionalized polymers are shown in the middle spectra in green. Unmodified polymers are shown in the bottom red spectra. Inset spectra show the maleimide region of the spectra from 6.0 – 6.8 ppm. $PDMA_{10}$-mal and $PDMA_{39}$-mal (green spectra **B** and **C**) exhibited polydispersity in SEC chromatograms (Figure S1), including possible polymers dimerized by a single bis-maleimide, and therefore spectra are provided without further assignment. Spectra were recorded on a 500 MHz Varian instrument in D_2O for $PDMA_{39}$ and in $CDCl_3$ for all other samples.

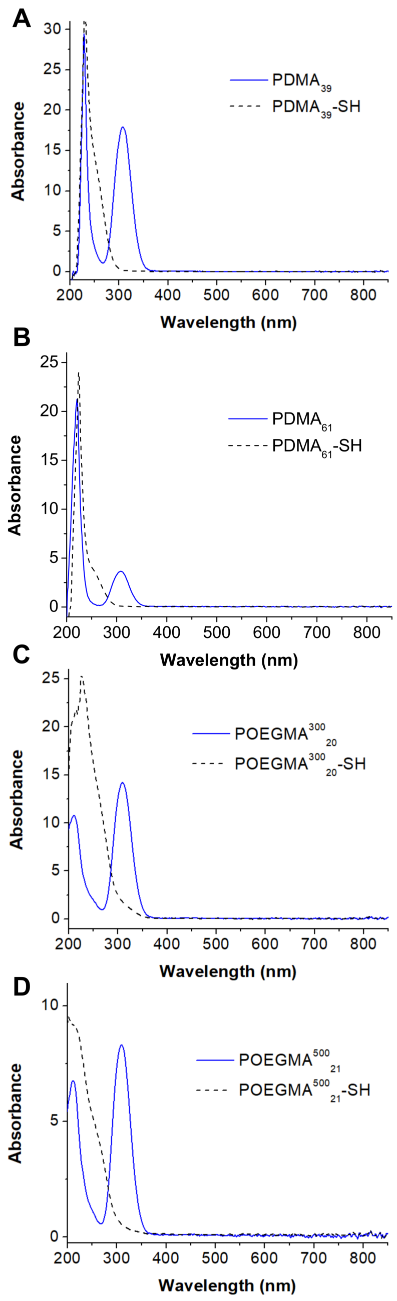

Figure S5. UV-Vis spectra of polymers before (blue traces) and after (black dashed traces) end-group cleavage with hydrazine. Samples were prepared by diluting 20-30X prior to acquisition and each polymer is prepared at the same dilution.

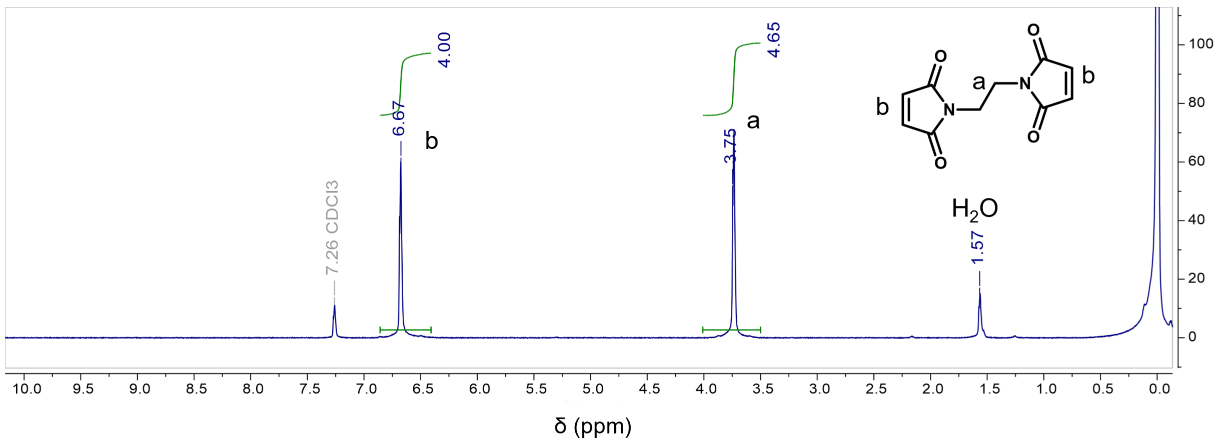

Figure S6. ^1H NMR spectrum measured in $CDCl_3$ measured on a Varian 500 MHZ instrument with the bis-maleimide linker (1) used to functionalize polymer thiol end groups.

Table S1. Exact fraction volumes of Gal3C[T243C] conjugates purified with SEC

Conjugate	Fraction	Volume Range (mL)
Gal3C[T243C]-P(OEGMA^{500})$_{21}$	A*	14.06 – 14.39*
	B*	14.39 – 14.72*
	C*	15.05 – 15.38*
	D*	15.71 – 16.04*
Gal3C[T243C]-PDMA$_{61}$	1*	14.86 – 15.19*
	2*	15.19 – 15.52*
	3*	15.52 – 15.85*
	4	15.85 – 16.18
Gal3C[T243C]-P(OEGMA^{300})$_{20}$	1	15.89 – 16.22
	2*	16.22 – 16.55*
	3*	16.55 – 16.88*
	4*	16.88 – 17.21*
	5	17.21 – 17.54
	6	18.20 – 18.53
Gal3C[T243C]-PDMA$_{10}$	a	16.55 – 16.88
	b*	16.88 – 17.21*
	c*	17.21 – 17.54*
	d*	17.54 – 17.87*
	e	18.20 – 18.53
Gal3C[T243C]-PDMA$_{39}$	A	15.05 – 15.38
	B	15.38 – 15.71
	C*	15.71 – 16.04*
	D*	16.04 – 16.37*
	E*	16.37 – 16.70*

*Denotes samples used for CD thermal melting analysis. All fractions were 330 μL

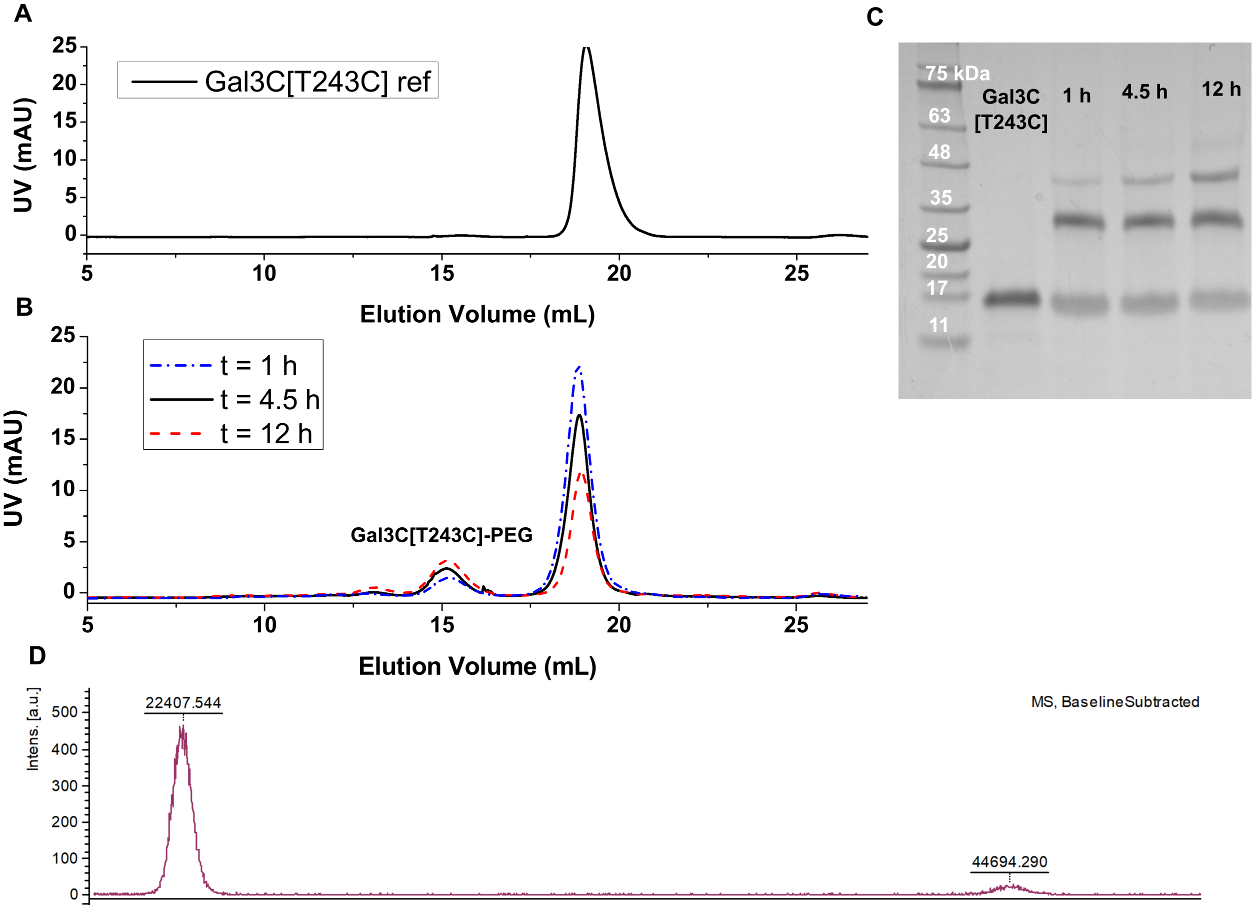

Figure S7. Determination of potential side products from the reaction between Gal3C[T243C] and 10 equivalents of 5k PEG-maleimide as monitored by SEC and SDS-PAGE. **A**) Reference Gal3C[T243C] SEC chromatogram. **B-C**) Reaction time points of 1, 4.5, or 12 h as shown in (**B**) superimposed chromatograms and (**C**) crude aliquots analyzed by SD-PAGE. **(D)** MALDI-TOF spectrum of 4.5 h sample time point showing masses (*m/z*) of 22.4 kDa and 44.7 kDa, at expected masses of Gal3C[T243C]-PEG conjugate and conjugate dimer, respectively. Spectrum was acquired in linear mode 20 – 50 kDa with a 10 mg / mL sinapinic acid matrix in 1:1 ACN: water (0.1% TFA).

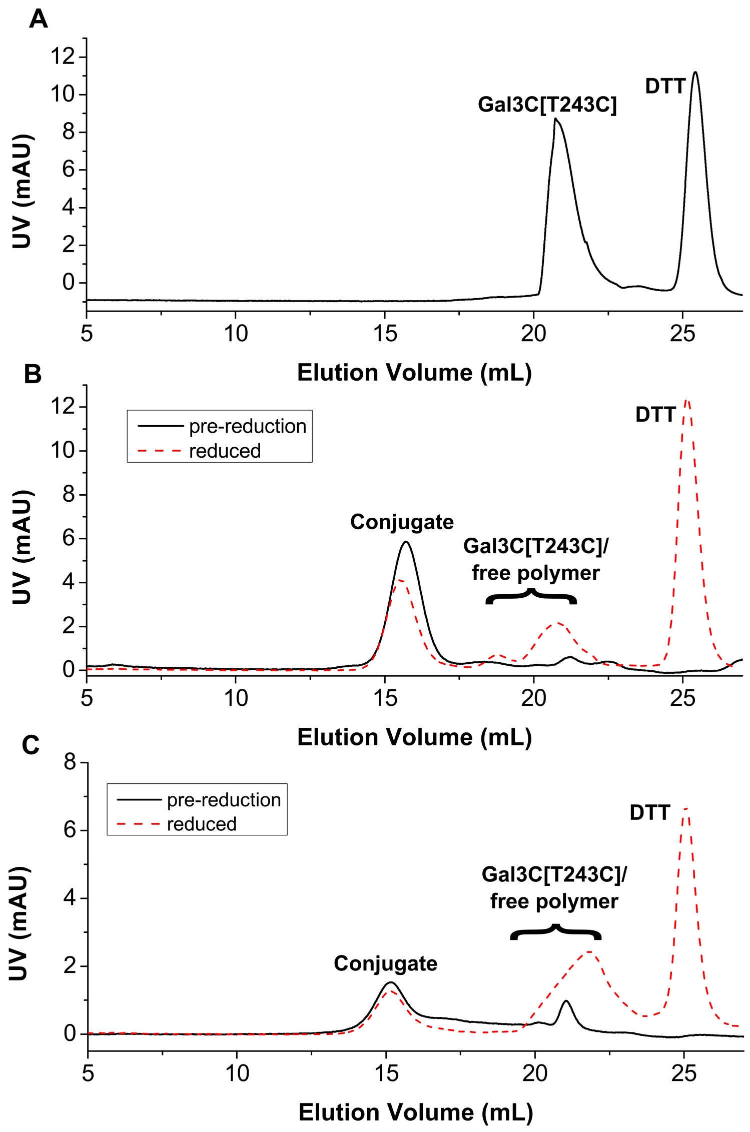

Figure S8. SEC-purified conjugates before (black traces) and after (red dash traces) reduction with DTT. **(A)** Reference chromatogram of Gal3C[T243C] with 10 mM DTT. **(B)** Gal3C[T243C]-$PDMA_{61}$ before and after DTT reduction. **(C)**. Gal3C[T243C]-$P(OEGMA^{500})_{21}$ before and after DTT reduction. Regions on **(B)** and **(C)** marked "Gal3C[T243C]/free polymer" are labeled according to Gal3C[T243C] elution times shown in **(A)** and elution time of free polymers (not shown).

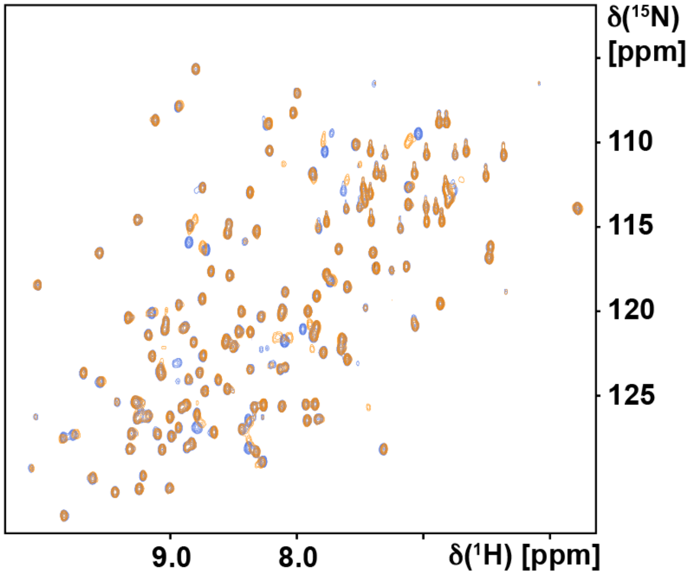

Figure S9. Superposition of [^{15}N,^{1}H]-HSQC spectra measured on a Bruker 800 MHz instrument at 30 °C of Gal3C[T243C] (blue) and Gal3C[T243C] conjugated to PEG (orange), as adapted with permission from reference 4.

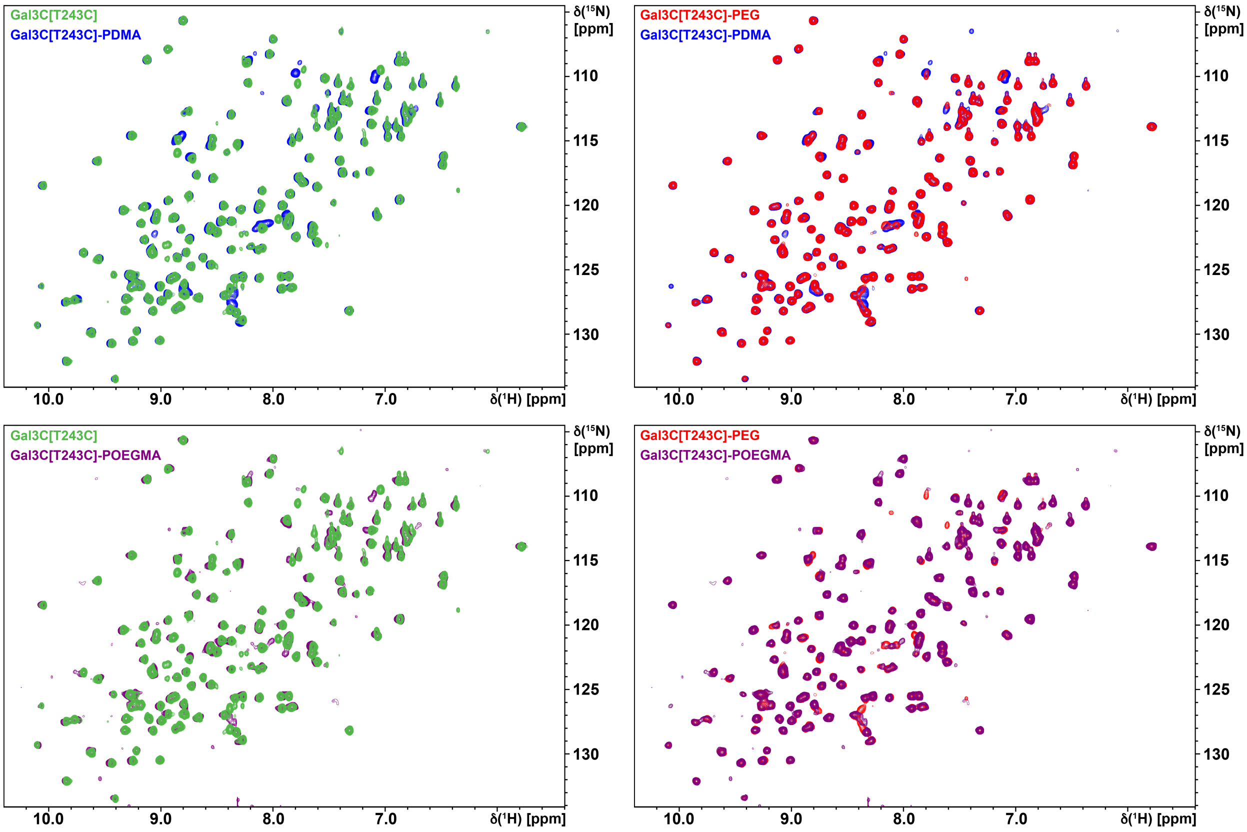

Figure S10. Superposition of [^{15}N,^{1}H]-HSQC spectra measured on a Bruker 800 MHz instrument at 30 °C of Gal3C[T243C] and Gal3C[T243C] conjugated to different polymers. The spectra of [u-^{15}N]-Gal3C[T243C] (green) and [u-^{15}N]-Gal3C[T243C]-PEG (red) is superimposed with the spectrum of [u-^{15}N]-Gal3C[T243C]-PDMA$_{21}$ (blue) or [u-^{15}N]-Gal3C[T243C]-P(OEGMA^{500})$_{21}$ (purple).

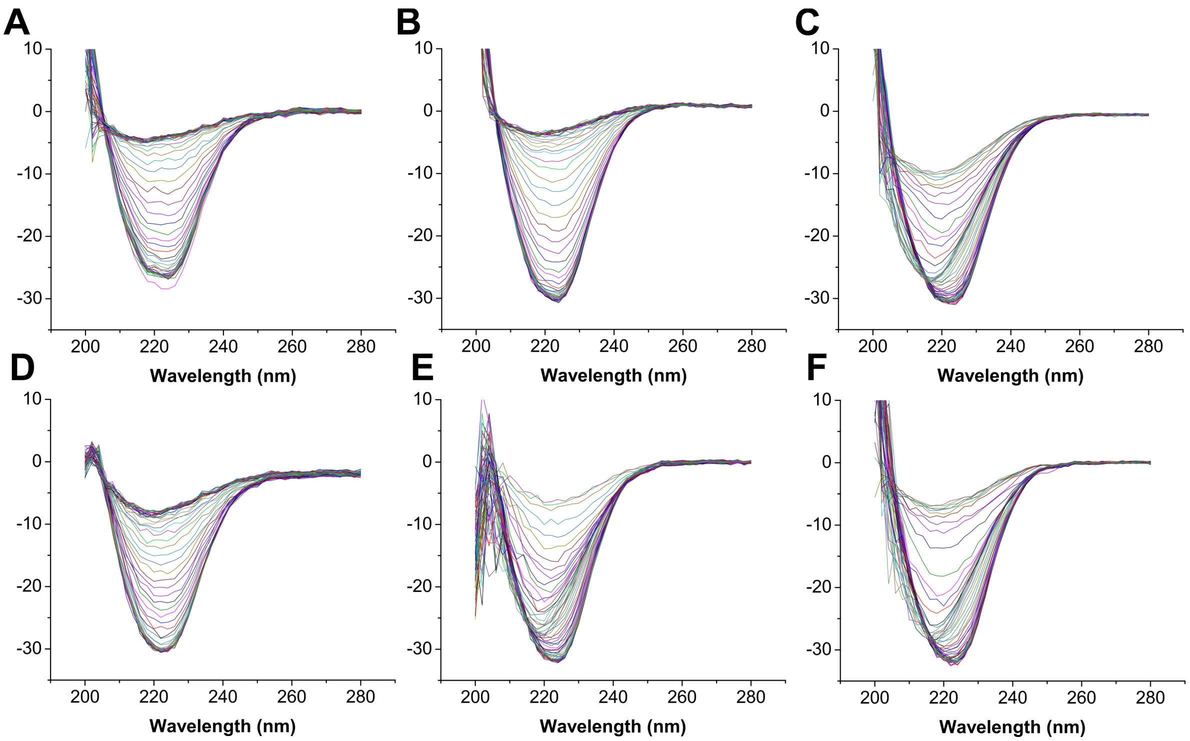

Figure S11. Superposition of full CD vs. wavelength thermal melting plots recorded from 30 to 90 °C, which were used to generate thermal melting curves shown in Figures 4 and 5. (**A**) Gal3C[T243C] spectra. (**B-F**) Melting plots Gal3C[T243C] conjugated with (**B**) P(OEGMA^{300})$_{20,}$ (**C**) P(OEGMA^{500})$_{21}$, (**D**) PDMA$_{10}$, (**E**) PDMA$_{39}$, and (**F**) PDMA$_{61}$.

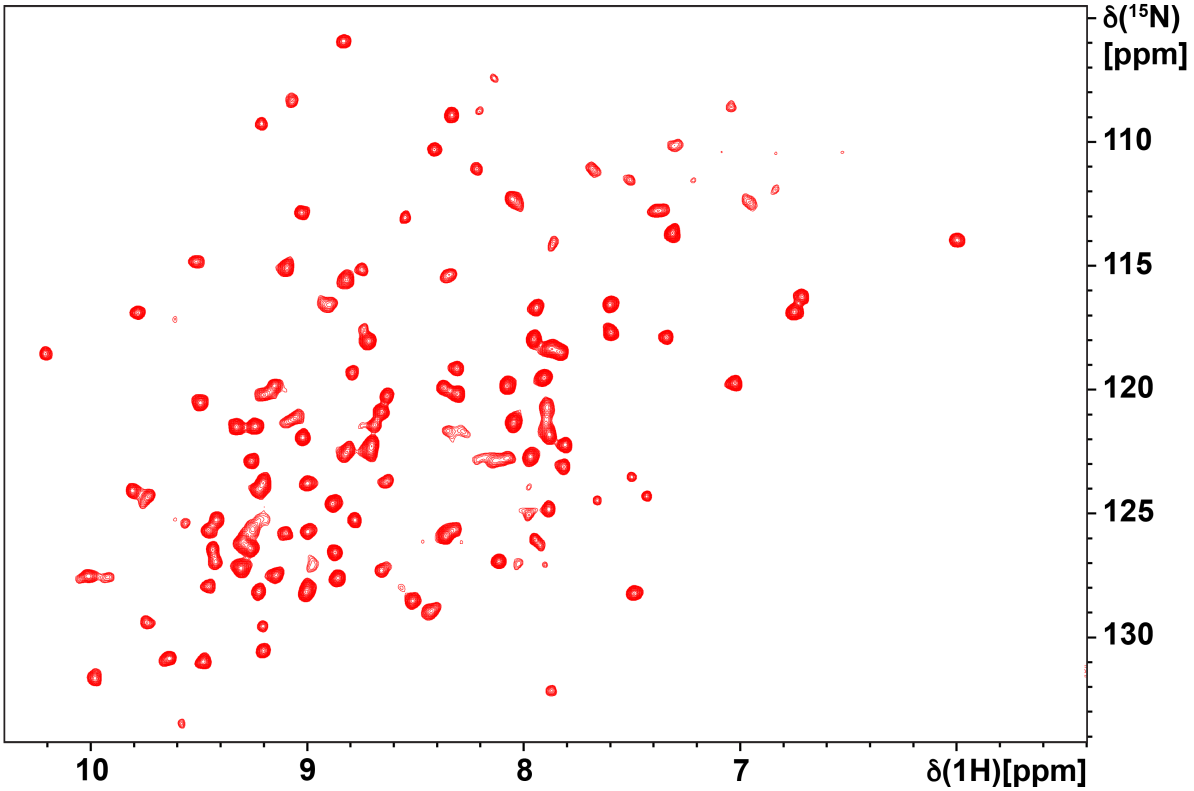

Figure S12. [^{15}N,^{1}H]-HSQC spectrum of [u-^{15}N]-Gal3C[T243C]-P(OEGMA^{500})$_{21}$ measured at 55 °C on a Bruker 800 MHz instrument.

References

(1) Sava, M. Preparation and characterization of bismaleimide monomers with various structures. *Designed Monomers and Polymers* **2013**, *16* (1), 14-24. DOI: 10.1080/15685551.2012.705485.

(2) Cava, M. P.; Deana, A. A.; Muth, K.; Mitchell, M. J. N-PHENYLMALEIMIDE. *Organic Syntheses* **1973**, *5*, 944.

(3) Shen, W.; Qiu, Q.; Wang, Y.; Miao, M.; Li, B.; Zhang, T.; Cao, A.; An, Z. Hydrazine as a Nucleophile and Antioxidant for Fast Aminolysis of RAFT Polymers in Air. *Macromolecular Rapid Communications* **2010**, *31* (16), 1444-1448, https://doi.org/10.1002/marc.201000154. DOI: https://doi.org/10.1002/marc.201000154 (acccessed 2022/12/10).

(4) Pritzlaff, A.; Ferré, G.; Mulry, E.; Lin, L.; Gopal Pour, N.; Savin, D. A.; Harris, M. E.; Eddy, M. T. Atomic-Scale View of Protein-PEG Interactions that Redirect the Thermal Unfolding Pathway of PEGylated Human Galectin-3. *Angewandte Chemie International Edition* **2022**, *61* (40), e202203784, https://doi.org/10.1002/anie.202203784. DOI: https://doi.org/10.1002/anie.202203784 (acccessed 2022/12/08).
