# Supplementary material for: Conserved Protein–Polymer Interactions across Structurally Diverse Polymers Underlie Alterations to Protein Thermal Unfolding
Source: ACS Cent Sci. 2023 Mar 14;9(4):685–95. doi: 10.1021/acscentsci.2c01522 (PMC10146661; doi:10.1021/acscentsci.2c01522)
Supplement: Supplementary file 2 — oc2c01522_si_002.pdf [file oc2c01522_si_002.pdf]

oc-2022-015226.R1

Name: Peer Review Information for "Conserved Protein-Polymer Interactions Across Structurally Diverse Polymers Underly Alterations to Protein Thermal Unfolding"

First Round of Reviewer Comments

Reviewer: 1

Comments to the Author

This is a very interesting paper which demonstrates the effect of the polymer on the protein stability. Although protein polymer conjugation is not a novel method, the novelty of this paper resides in the high-resolution NMR characterization and interpretation.

I have some comments regarding the protein polymer conjugations:

1. The authors mention that all polymers were monodispersed (Figure S1). I am not sure this correct. According to Figure S1, molecular weight distributions are relatively broad. In fact, after reaction with bismaleimide, B is very broad and shows the presence of shoulder, as D. What is the reaction yield of this conjugation. It will be interesting to superimpose the before and after modification for each polymer on one graph. This will show if there are some side reactions.

according to figure s3, the authors did not include a potential coupling polymer by reaction of two thiols on one maleimide.

the authors used SEC and GPC. SEC should be used instead of GPC.

The PAGEs show the presence of unconjugated proteins (Figure 4 and 5) as this affect the HSQC spectra, and therefore the interpretation.

Reviewer: 2

Comments to the Author

Very interesting manuscript. The work clearly shows significant protein stabilization through coupling to these polymers. I am intrigued by the intermediate that forms and expect future characterization. To note, I am not a polymer chemist and was largely unaware of the significance of the use of these polymers and biological problems, for example many PEGs give rise to antibodies. To that end, on p1 second column line 44, perhaps a statement about why these particular PEGs are biomedically relevant could be included. This could strengthen the introduction as the research points to important biomedical possibilities of coupling such polymers to enhance efficacy of protein drugs.

The following are some issues I have and suggestions for editing.

Major:

p5 line 17-19 it says peaks in the maleimide were observed, and just above stated as at 6.62 ppm. In Fig S4C I cannot see such a peak. But there is a peak near 6.3 and in other spectra 6.2 to 6.3 ppm. Is this the relevant peak? If so, make clearer or explain.

Figure 3 and reference to text. The text states on bottom of p6 “In contrast to the Gal3C[T243C] conjugate with PDMA, we observed more significant line broadening for a wider range of residues in NMR data of the conjugate with POEGMA (Figure 3 and Figure S9).” (that is possibly Figure S10). However, if I understand Figure 3 A) PEG B) POMEGA C) PMDA. The histogram to me shows more extensive broadening in C) compared to B) saying PMDA causes more shift/broadening changes? The largest shifting peak in B) is not observed in C) because the signal in C) is completely broadened. Even the mapping on F) looks more extensive than in E). Could this be mislabeling? Or I need some clarification/convincing. Also in Figure 3 state it is the difference of the conjugated from Gal3C[T243C].

p8 first column lines 39 and near. I do not really understand “collapsed” and “extended” and this needs better explanation as this paragraph says the more extensive perturbations are due to an extended conformer. Why is peak doubling expected for a collapsed model? The broadening could be exchange between two "distinct" states (collapsed or extended). Why assume slow exchange between these proposed states?

Top of p8 second column, I think should be Figure 3 if referring to NMR data. Despite my comment above, for the sentence “…proteinpolymer interactions, as observed by NMR (Figure 2), lead to the formation of a clear thermal unfolding intermediate state.” I cannot see how you can connect the NMR features (broadening and chemical shift) with the observed CD unfolding data that definitely shows an intermediate. The statements may need rewriting.

I think the conclusion can be rewritten and shortened. It is mostly a summary and not a conclusion; except perhaps the last two paragraphs.

Minor:

Suggest everywhere to write Kd as capital K and capital subscripted D

I think where POMEGA is in the context of one of the polymers to write as P(OMEGA) eg P(OMEGA)50021, also in the supp, eg Figure S3 P(OMEGA)30020

Within the legends be consistent in format eg Figure 3 (D-F)

On p6 second column concerning thiols in the polymer; are these contaminating polymers, as the description of the polymers does not indicate any thiol.

On p5 last paragraph column 2, “stable-isotope” is not needed

On p7 second column line 37, I think it is Figure S11

Reviewer: 3

Comments to the Author

This manuscript describes a biophysical approach to understanding how polymer conjugation can stabilize proteins, a strategy that is often used in pharmaceutical and industrial applications. PEG is frequently used for this purpose, but its linear architecture imposes some limitations and some patients have developed sensitivity to it. Therefore, it is desirable to test whether other polymers can proteins, and to understand the molecular basis of this effect. Here the test protein is human Gal3. The systematic approach used here, with polymers with different chemical functionality and degree of branching, is a strength. The paper is well written and the results are clearly presented in general. However, I think the analysis could be strengthened by more quantitative discussion of the NMR data. The chemical shift perturbation, which shows which residues are directly impacted by polymer conjugation is clear, but the interpretation of the “broadening index” is less so. Can the authors comment on what this means on the molecular level? What kind of motion is this broadening describing, and on what timescale? I am not asking for additional dynamics experiements to be performed (although that could be an interesting follow-up study), just a more detailed discussion even if it is somewhat speculative at this point. Also, can the authors comment on the transition from two-state to three-state melting curves in Figure 5? What do you think the intermediate is, and how is the polymer contributing to its formation?

Author's Response to Peer Review Comments:

Dear Dr. Editor,

In the attachments, we submit our point-by-point response to the reviewers and revisions to the manuscript “Conserved Protein-Polymer Interactions Across Structurally Diverse Polymers Underly Alterations to Protein Thermal Unfolding” by Pritzlaff et al.

We were encouraged by the overall positive comments of all three reviewers. We believe we have been able to accommodate suggestions and questions from all the reviewers and have incorporated the requested changes in our revised manuscript. We hope the revised manuscript is acceptable for publication. Please do not hesitate to reach out if we can be of assistance in addressing any questions.

Best Regards,

Matthew Eddy

ACS Central Science Manuscript ID oc-2022-015226
Response to Reviewers

Comments from the reviewers are given below in black, and our point-by-point response is provided below each comment in blue.

Revisions are indicated using a yellow background in the revised manuscript, per instructions from the editorial office.

Reviewer #1:

This is a very interesting paper which demonstrates the effect of the polymer on the protein stability. Although protein polymer conjugation is not a novel method, the novelty of this paper resides in the high-resolution NMR characterization and interpretation.

First, we appreciate the reviewer's positive assessment of our work. We also appreciated the questions and comment, which allowed us to revise and strengthen the manuscript

I have some comments regarding the protein polymer conjugations:
1. The authors mention that all polymers were monodispersed (Figure S1). I am not sure this correct. According to Figure S1, molecular weight distributions are relatively broad. In fact, after reaction with bismaleimide, B is very broad and shows the presence of shoulder, as D. What is the reaction yield of this conjugation. It will be interesting to superimpose the before and after modification for each polymer on one graph. This will show if there are some side reactions.

We appreciate the reviewer's comments. Our original text on page 3 was intended to convey that synthesized polymers were monodispersed prior to end-group functionalization. The revised text on page 3 and new sub-heading above this paragraph clarify this point.

We observed that some polymers appeared polydisperse after end-group functionalization with bismaleimide. To more clearly present these observations, we included a new Figure S1, which shows superimposed size exclusion chromatograms of the polymer before and after functionalization. In the new Figure S1, chromatograms of $PDMA_{10}$ and $PDMA_{39}$ (Figure S1A and B) show the presence of side products, which are discussed in detail in the main text on Page 4. Because only the monomeric polymer could react with Gal3C through thiol-Michael addition or, to a lesser extent, disulfide coupling, reactions with these multi-modal polymer products resulted in a single addition of monomeric polymer with Gal3C. Our results from SDS-PAGE and SEC of the protein-polymer conjugates for these two species (Figure 4B,E for PDMA10 and Figure 4C,F for PDMA39) confirm that a single addition of monomeric polymer occurred, as documented by the relative shifts in the SDS-PAGE gels or SEC elution time, respectively.

The yield of functionalization, i.e., yield of polymer successfully reacted with the maleimide, is described in the revised text on Pages 3 (right column, bottom) and 5 (left

column) as ranging from 12-20% for POEGMA and 8% for PDMA. We consider the POEGMA integration to be more reflective of true functionalization yield due to the more precise integration of the spectrum for this conjugate. Despite the low conversion rate, we were still able to generate sufficient conjugates from the excess equivalents of polymer reacted with protein.

according to figure s3, the authors did not include a potential coupling polymer by reaction of two thiols on one maleimide.

The polymers characterized with ^{1}H-NMR in Figure S3 were primarily monomeric, as indicated by SEC analysis (Figure S1). However, it is difficult to detect the presence of dimers of larger polymers using ^{1}H-NMR, as they likely exhibit similar chemical shifts. The ^{1}H-NMR data for the PDMA polymers in Figure S4 include a mixture of polydisperse products, as noted by the reviewer. Because we could not confirm assignments, we provided spectra without a suggested assignment. The caption of Figure S4 has been revised to acknowledge the possibility of dimers as one of the observed components.

the authors used SEC and GPC. SEC should be used instead of GPC.

We have revised the text to use SEC consistently throughout.

The PAGEs show the presence of unconjugated proteins (Figure 4 and 5) as this affect the HSQC spectra, and therefore the interpretation.

The SDS-PAGE analysis shown in Figure 2D-E suggests the samples appear to contain approximately 70% conjugated protein. However, the results from the control conjugate reduction experiment shown in Figure S8 indicate that the reducing conditions used in the SDS-PAGE analysis may underestimate the amount of conjugated protein by 10-20% of the total protein content. This interpretation is supported by the HSQC NMR data. In the case of a mixture of conjugated and unconjugated protein, the NMR spectra would show two sets of signals for residues that exhibit chemical shift perturbations for the conjugated protein, i.e. peak doubling would be observed throughout the spectra. Since we do not observe these features, we can conclude that any unconjugated protein present is below the detection limit of our experiment, amounting to approximately less than 10% of the total protein fraction. The relevant text on pages 5 and 6 has been revised to reflect these observations and provide a more comprehensive explanation.

Reviewer #2:

Very interesting manuscript. The work clearly shows significant protein stabilization through coupling to these polymers. I am intrigued by the intermediate that forms and expect future characterization.

We appreciate the reviewer's positive assessment of our work and the questions and comments.

To note, I am not a polymer chemist and was largely unaware of the significance of the use of these polymers and biological problems, for example many PEGs give rise to antibodies. To that end, on p1 second column line 44, perhaps a statement about why these particular PEGs are biomedically relevant could be included. This could strengthen the introduction as the research points to important biomedical possibilities of coupling such polymers to enhance efficacy of protein drugs.

We appreciate the reviewer's input on this matter and have taken it into consideration. As a result, the text on page 1 has been expanded to provide more comprehensive background information on the significance of PDMA and POEGMA in biomedical fields. This includes discussion of the reported benefits such as enhanced plasma circulation times and improved ability to evade the immune system.

The following are some issues I have and suggestions for editing.

Major:

p5 line 17-19 it says peaks in the maleimide were observed, and just above stated as at 6.62 ppm. In Fig S4C I cannot see such a peak. But there is a peak near 6.3 and in other spectra 6.2 to 6.3 ppm. Is this the relevant peak? If so, make clearer or explain.

On page 5, the text has been revised to indicate the presence of a maleimide peak was observed at 6.67 ppm for $PDMA_{39}$ in the ^1H NMR spectrum, however, it was not resolved in the spectrum of the $PDMA_{10}$ polymer. The peaks observed near 6.2 ppm are not attributed to the maleimide. We proceeded with conjugation using polydispersed polymer samples based on the fact that only monomeric polymer products contain the maleimide group, which is necessary for reaction with the protein. Additionally, a large excess of polymer (10 equivalents) was reacted with the protein in an effort to overcome the low functionalization of the target end group.

Figure 3 and reference to text. The text states on bottom of p6 "In contrast to the Gal3C[T243C] conjugate with PDMA, we observed more significant line broadening for a wider range of residues in NMR data of the conjugate with POEGMA (Figure 3 and Figure S9)." (that is possibly Figure S10). However, if I understand Figure 3 A) PEG B) POMEGA

C) PMDA. The histogram to me shows more extensive broadening in C) compared to B) saying PMDA causes more shift/broadening changes? The largest shifting peak in B) is not observed in C) because the signal in C) is completely broadened. Even the mapping on F) looks more extensive than in E). Could this be mislabeling? Or I need some clarification/convincing. Also in Figure 3 state it is the difference of the conjugated from Gal3C[T243C].

We appreciate the reviewer's observations. The confusion arose due to a mistake in the labeling of the panels in Figure 3. This was caused by an error during the revision of the figure order from an earlier version of the manuscript, where the corresponding figure legend was not updated. We have corrected this mistake. Additionally, we have thoroughly checked the figure legends in the other NMR data figures and found them to be correct. Furthermore, we have verified the accuracy of the revised Figure 3 legend by referencing the original data.

p8 first column lines 39 and near. I do not really understand "collapsed" and "extended" and this needs better explanation as this paragraph says the more extensive perturbations are due to an extended conformer. Why is peak doubling expected for a collapsed model? The broadening could be exchange between two "distinct" states (collapsed or extended). Why assume slow exchange between these proposed states?

In this context, the terms "collapsed" and "extended" refer to the conformation of the polymer backbone, specifically its hydrophobic main chain, in solution. PEG and PDMA are linear polymers that typically exhibit an extended, random coil-like conformation in solution. Conversely, POEGMA, with its numerous branches or side chains, can adopt either an extended, coil-like backbone conformation or a more compact, collapsed conformation, depending on factors such as the length and chemical structure of its side chains and the composition of the solution used to solubilize the polymer.

The NMR data for Gal3C conjugated to $P(OEGMA^{500})_{21}$ suggest a more extended conformation of the polymer, as changes in the HSQC NMR data were spread over a wider region of the protein surface. Peak doubling would likely not be expected for a collapsed model. Rather, we would expect to observe a set of chemical shift perturbations that are more locally confined near the conjugation site. We recognize multiple potential contributions to peak broadening for the conjugated proteins, including fluctuations in protein-polymer interactions due to changes in the polymer conformation. We agree that such fluctuations do not necessarily occur on a slow timescale and further data is likely needed to precisely define these timescales in a future study. We have revised and expanded the relevant text to more clearly explain these points and address the reviewer's concerns.

Top of p8 second column, I think should be Figure 3 if referring to NMR data. Despite my comment above, for the sentence "…proteinpolymer interactions, as observed by NMR (Figure 2), lead to the formation of a clear thermal unfolding intermediate state." I

cannot see how you can connect the NMR features (broadening and chemical shift) with the observed CD unfolding data that definitely shows an intermediate. The statements may need rewriting.

We concur with the reviewer's comments and have revised the text to highlight the contrast in hydrophobicity and lengths between the compared polymers and their different effects on the thermal unfolding of the conjugated proteins.

I think the conclusion can be rewritten and shortened. It is mostly a summary and not a conclusion; except perhaps the last two paragraphs.

We have revised the conclusion to shorten it and remove redundant information.

Minor:

Suggest everywhere to write Kd as capital K and capital subscripted D

We have made the requested revision.

I think where POMEGA is in the context of one of the polymers to write as P(OMEGA) eg P(OMEGA)50021, also in the supp, eg Figure S3 P(OMEGA)30020

We have revised the text to use consistent nomenclature throughout. Specifically, the section describing the HSQC NMR data and also relevant sections in the Supporting Information have been revised to specify $PDMA_{61}$ and $P(OEGMA^{500})_{21}$.

Within the legends be consistent in format eg Figure 3 (D-F)

We have corrected the figure legend formatting for Figure 3, panels D-F.

On p6 second column concerning thiols in the polymer; are these contaminating polymers, as the description of the polymers does not indicate any thiol.

The polymer-thiol is an intermediate in the polymer functionalization reaction, which is also a side product since not all of the polymer-thiol is reacted with the maleimide linker. A revised Polymer End Group Modification section on page 3 now discusses this more thoroughly.

On p5 last paragraph column 2, "stable-isotope" is not needed

We have made the requested revision.

On p7 second column line 37, I think it is Figure S11

We thank the reviewer for pointing out this oversight and have made the correction.

Reviewer #3:

This manuscript describes a biophysical approach to understanding how polymer conjugation can stabilize proteins, a strategy that is often used in pharmaceutical and industrial applications. PEG is frequently used for this purpose, but its linear architecture imposes some limitations and some patients have developed sensitivity to it. Therefore, it is desirable to test whether other polymers can proteins, and to understand the molecular basis of this effect. Here the test protein is human Gal3. The systematic approach used here, with polymers with different chemical functionality and degree of branching, is a strength. The paper is well written and the results are clearly presented in general.

We thank the reviewer for their positive assessment of our work and for the suggestions for further improving the text.

However, I think the analysis could be strengthened by more quantitative discussion of the NMR data. The chemical shift perturbation, which shows which residues are directly impacted by polymer conjugation is clear, but the interpretation of the "broadening index" is less so. Can the authors comment on what this means on the molecular level? What kind of motion is this broadening describing, and on what timescale? I am not asking for additional dynamics experiements to be performed (although that could be an interesting follow-up study), just a more detailed discussion even if it is somewhat speculative at this point.

The term "broadening index" was used to describe the combined effects on the observed line broadening of conjugated Gal3C, including both indirect impacts on protein conformational dynamics and direct protein-polymer interactions. The difficulty in experimentally separating these contributions led to using this term to avoid overinterpretation of the data. Despite this, the analysis can still be expanded within the limitations of the study.

Line broadening was observed to be specific to individual residues, not uniformly observed for the entire protein, indicating that local changes in protein dynamics caused by conjugation were responsible, rather than significant changes in the diffusion of the conjugated protein.

The changes in line broadening observed in HSQC spectra Gal3C-polymer conjugates can be likely attributed to conformational dynamics of the backbone amide groups as our analysis takes into account the contributions from both the ω_{15N} and ω_{1H} dimensions to the modulation of signal intensities in the 2D spectra. This effect is observed in a subset of residues close to the conjugation site in spectra of Gal3C-PDMA and more extensively throughout the protein in spectra of Gal3C-POEGMA (as shown in Figure 3). Both the POEGMA and PDMA polymers are of similar molecular sizes and are chemically conjugated to the same residue in Gal3C. The different patterns of line broadening observed for the two conjugates are thus likely a result of differences in protein-polymer

interactions due to distinct chemical structures of the monomers and differences in polymer branching. Similar observations in HSQC spectra of globular proteins are typically attributed to fluctuations in the protein backbone on millisecond to microsecond time scales. Because we could not delineate all potential contributions to broadening, we focused on analyzing the patterns of line broadening for the different conjugates to assess if evidence for different patterns of protein-polymer interactions was present. We have included these insights in the revised conclusion section.

Also, can the authors comment on the transition from two-state to three-state melting curves in Figure 5? What do you think the intermediate is, and how is the polymer contributing to its formation?

In this study, CD spectra recorded within the temperature range of the intermediate state showed results consistent with a globally folded protein with primarily beta sheet secondary structure (Figure S11). The global fold of the intermediate is thus likely similar to the global fold of the conjugated protein at lower temperature, but not identical as we observe a shift in the λ_{min} value from 222 nm to 218 nm during the formation of the intermediate state.

The folded state of Gal3C[T243C]-P(OEGMA^{500})$_{21}$ at higher temperature was also confirmed by 2D HSQC spectra at 55 ºC, the highest temperature we could operate the NMR cryoprobe. Although this temperature is below the temperature range for the formation of the intermediate (around 65 ºC to 75 ºC), the NMR data support the CD measurements and shows that the conjugated protein remains folded up to temperatures close to the formation of the intermediate. Please refer to the new Figure S12.

The transition from a two-state to three-state melting curve in Figure 5 panel G resembles what was seen in our previous study of Gal3C[T243C] conjugated with PEG$_{4.5k}$,[1] which showed the formation of an intermediate state at increased temperature. In that study, we attributed intermediate formation to sustained protein-polymer interactions at elevated temperatures. However, we were limited to exploring temperatures below 55 ºC by NMR due to limitations in instrumentation. In future studies, we hope to access more specialized NMR instrumentation that are compatible with exploring higher temperatures.

Added information on these observations can be found on page 8 of the corresponding revised text.

1. [1] Pritzlaff, Amanda, et al. *Angewandte Chemie International Edition* 61.40 (2022): e202203784.

oc-2022-015226.R2

Name: Peer Review Information for "Conserved Protein-Polymer Interactions Across Structurally Diverse Polymers Underly Alterations to Protein Thermal Unfolding"

Second Round of Reviewer Comments

Reviewer: 2

Comments to the Author

The manuscript is much improved and is well written.

Reviewer: 3

Comments to the Author

The authors have addressed my concerns about the previous manuscript.

Author's Response to Peer Review Comments:

We have removed the highlighting to the main text and SI document and are uploading clean versions. We have also provided credit to Figure 2 to the panels which were adapted from Reference 33. I have permission to use these images as I am the contributor of them from my group's previous publication.
